# Molecular profiling of hormone receptor-positive, HER2-negative breast cancers from patients treated with neoadjuvant endocrine therapy in the CARMINA 02 trial (UCBG-0609)

**DOI:** 10.1186/s13045-018-0670-9

**Published:** 2018-10-11

**Authors:** Xu Liang, Adrien Briaux, Véronique Becette, Camille Benoist, Anais Boulai, Walid Chemlali, Anne Schnitzler, Sylvain Baulande, Sofia Rivera, Marie-Ange Mouret-Reynier, Laurence Venat Bouvet, Thibaut De La Motte Rouge, Jérôme Lemonnier, Florence Lerebours, Céline Callens

**Affiliations:** 1 Key Laboratory of Carcinogenesis and Translational Research (Ministry of Education/Beijing), Department of Breast Oncology, Peking University Cancer Hospital & Institute, Beijing, China; 2 Pharmacogenomic Unit, Department of Genetics, Curie Institute, PSL Research University, Paris, France; 3 Department of Biopathology, Curie Institute, René Huguenin Hospital, Saint-Cloud, France; 4 Institut Curie Genomics of Excellence (ICGex) Platform, Curie Institute, PSL Research University, Paris, France; 5 Department of Radiotherapy, Gustave Roussy, Villejuif, France; 6 Department of Medical Oncology, Centre Jean Perrin, Clermont-Ferrand, France; 7 Department of Medical Oncology, CHU, Limoges, France; 8 Department of Medical Oncology, Centre Eugene Marquis, Rennes, France; 9 R&D Unicancer, UCBG, Paris, France; 10 Department of Medical Oncology, Curie Institute, René Huguenin Hospital, Saint-Cloud, France

**Keywords:** Immunity, Lipid metabolism, Somatic mutation, TILs, RNA sequencing, Targeted NGS, Endocrine therapy, Breast cancer

## Abstract

**Background:**

Postmenopausal women with large, hormone receptor (HR)-positive/HER2-negative and low-proliferative breast cancer derived a benefit from neoadjuvant endocrine therapy (NET) in the CARMINA02 trial. This study was designed to correlate gene expression and mutation profiles with both response to NET and prognosis.

**Methods:**

Gene expression profiling using RNA sequencing was performed in 86 pre-NET and post-NET tumor samples. Targeted next-generation sequencing of 91 candidate breast cancer-associated genes was performed on DNA samples from 89 patients. Molecular data were correlated with radiological response and relapse-free survival.

**Results:**

The transcriptional profile of tumors to NET in responders involved immune-associated genes enriched in activated Th1 pathway, which remained unchanged in non-responders. Immune response was confirmed by analysis of tumor-infiltrating lymphocytes (TILs). The percentage of TILs was significantly increased post-NET compared to pre-NET samples in responders (*p* = 0.0071), but not in non-responders (*p* = 0.0938). Gene expression revealed that lipid metabolism was the main molecular function related to prognosis, while PPARγ is the most important upstream regulator gene. The most frequently mutated genes were *PIK3CA* (48.3%), *CDH1* (20.2%), *PTEN* (15.7%), *TP53* (10.1%), *LAMA2* (10.1%), *BRCA2* (9.0%), *MAP3K1* (7.9%), *ALK* (6.7%), *INPP4B* (6.7%), *NCOR1* (6.7%), and *NF1* (5.6%). Cell cycle and apoptosis pathway and PIK3CA/AKT/mTOR pathway were altered significantly more frequently in non-responders than in responders (*p* = 0.0017 and *p* = 0.0094, respectively). The average number of mutations per sample was significantly higher in endocrine-resistant tumors (2.88 vs. 1.64, *p =* 0.03), but no difference was observed in terms of prognosis. *ESR1* hotspot mutations were detected in 3.4% of treatment-naive tumors.

**Conclusions:**

The Th1-related immune system and lipid metabolism appear to play key roles in the response to endocrine therapy and prognosis in HR-positive/HER2-negative breast cancer. Deleterious somatic mutations in the cell cycle and apoptosis pathway and PIK3CA/AKT/mTOR pathway may be relevant for clinical management.

**Trial registration:**

This trial is registered with ClinicalTrials.gov (NCT00629616) on March 6, 2008, retrospectively registered.

**Electronic supplementary material:**

The online version of this article (10.1186/s13045-018-0670-9) contains supplementary material, which is available to authorized users.

## Background

Hormone receptor-positive and human epidermal growth factor receptor 2 (HER2)-negative breast cancer represents approximately 70% of all breast cancers [1]. Endocrine therapy prevents cancer recurrence in the majority of patients, but a significant proportion of patients exhibit de novo or acquired resistance and up to 20% of patients subsequently relapse and die from metastatic disease [2]. The development of gene expression profiling facilitates several multi-gene assays, providing predictive and prognostic information in addition to useful clinicopathological characteristics. Current genomic tests for breast cancer, such as OncotypeDx or MammaPrint, are useful for prognosis and to guide adjuvant chemotherapy. Patients in the low-risk category according to BCI, EndoPredict, or Prosigna have been shown to present an extremely low risk of distant late recurrence, therefore allowing extensive adjuvant hormonal therapy to be avoided. However, these multi-gene assays were not designed for prediction, particularly of endocrine sensitivity [3]. A few studies have tested multi-gene assays or Ki-67-associated genes to predict response to neoadjuvant endocrine therapy (NET), but the results frequently lack power due to the small number of patients [4–9]. A four-gene model was recently proposed to predict response to aromatase inhibitors [10], consisting two genes before treatment and two proliferation genes after 2 weeks of therapy, but an additional biopsy within a period of 2 weeks may be difficult to apply in everyday practice.

The neoadjuvant setting provides a unique opportunity to investigate the mechanisms associated with endocrine sensitivity. A more powerful and informative approach consists of comparing sequential biopsies from the same patients before and after NET. The CARMINA02 trial reported the efficacy of anastrozole or fulvestrant as neoadjuvant therapy in postmenopausal women with hormone receptor (HR)-positive/HER2-negative breast cancer. Comparable good clinical response and survival rates in both arms showed that NET could be a treatment option in this breast cancer population. The usual biomarkers, Ki-67, ER Allred score, and Preoperative Endocrine Prognostic Index (PEPI) score, were investigated in this trial; neither baseline Ki-67 level nor a high ER Allred score were predictive of response [11].

In the present study, we performed RNA and DNA analysis, and immunohistochemistry (IHC) of tumor-infiltrating lymphocytes (TILs) on samples obtained from patients included in the CARMINA02 trial. Combined analysis of genomic profiling and completed clinical outcomes may provide insight into the underlying mechanisms of endocrine sensitivity.

## Methods

### Patients

The CARMINA 02 phase 2, multicenter, open-label, non-comparative, randomized trial was designed to evaluate, side by side, the efficacy of anastrozole and fulvestrant as NET in postmenopausal women with operable breast cancer. A total of 116 patients (59 in the anastrozole arm and 57 in the fulvestrant arm) were randomized (1:1) to receive either anastrozole (1 mg daily) or fulvestrant (500 mg with a loading dose on day 1, 15, and 29 for the first month and then every 4 weeks). The study was conducted in accordance with the Declaration of Helsinki and Good Clinical Practice guidelines. All patients provided their written informed consent. The study was authorized by the French National Agency for Medicines and Health Products Safety and was approved by the Ile de France VIII ethics committee. The consent form indicated that “medical information created by this study may become part of your medical record.” The patients were informed that their protected health information may be shared with the sponsors of the study. Fifty-seven patients were analyzed for the primary endpoint in each arm.

### Tumor samples and response assessment

Tumor samples for RNA analysis were core biopsies performed before treatment and surgical specimens obtained after NET. Samples were snap-frozen in liquid nitrogen and formalin-fixed paraffin-embedded (FFPE). FFPE blocks were taken for staining with hematoxylin and eosin for assessment of cellularity and percentage of invasive cancer by the pathologist. A total of 86 frozen samples from 55 patients (including 31 paired samples and 24 pretreatment-only samples) were used for RNA sequencing. Eighty-nine DNA samples were obtained from FFPE blocks from macro-dissection of post-treatment surgical specimens for targeted next-generation sequencing (NGS) analysis. Clinical and radiological response was determined by palpation and measuring dynamic changes in tumor size on breast ultrasound and magnetic resonance imaging (MRI), respectively. Objective tumor responses and pathological response were defined as previously described [11]. Relapse-free survival (RFS) was a secondary endpoint of the CARMINA02 trial. With a median follow-up of 65.7 months (95%CI 60.1–64.6 months), 19 events (2 patients of loco-regional relapses and 12 distant relapses with 3 deaths from breast cancer, 1 death from heart failure, and 4 deaths from unknown cause) were observed in 116 patients. Among the 55 patients with RNA sequencing, 7 patients presented breast cancer relapsed and 45 patients presented no relapse. Data from 3 patients who died from unknown causes or another cancer were excluded from the prognostic analysis. A total of 16 events (1 patient of loco-regional relapses and 10 distant relapses with 2 deaths from breast cancer, 1 death from heart failure, and 4 deaths from unknown cause) were observed among the 89 patients with targeted NGS data.

### Tumor-infiltrating lymphocyte evaluation

FFPE sections of pretreatment core-cut biopsies and post-treatment surgical specimens were H&E-stained, and the presence or absence of detectable TILs pre- and post-NET was assessed by a pathologist according to the international TILs working group guidelines [12]. Stromal TILs were determined as the percentage of immune cells in stromal tissue of tumors presenting a mononuclear cell infiltrate. The number of TILs was analyzed as a continuous variable.

### RNA sequencing, transcriptome read alignment, and data analysis

RNA was extracted and prepared from frozen tissue with the Qiagen RNeasy Mini Kit (Cat. 74104), according to the manufacturer’s protocol. All samples were subjected to quality control on a Bioanalyzer instrument and only RNA with RIN (RNA Integrity Number) > 7 was used for sequencing. RNA sequencing libraries were prepared from 1 μg of total RNA using the Illumina TruSeq Stranded mRNA Library preparation kit that can be used to perform strand-specific sequencing. A first step of polyA selection using magnetic beads was performed to focus sequencing on polyadenylated transcripts. After fragmentation, cDNA synthesis was performed and the resulting fragments were used for dA-tailing and then ligated to TruSeq indexed adapters. PCR amplification was then performed to create the final cDNA library. After qPCR quantification, sequencing was carried out using 2 × 100 cycles (paired-end reads, 100 nucleotides) on a Illumina HiSeq2500 instrument (high-output flow cells) to obtain about 200 million paired reads per sample. Reads were aligned to the human reference genome hg19/GRCh37 using TopHat2 v2.0.6 [13] with the following parameters: global alignment, no mismatch in the 22 bp seed, up to three mismatches in the read, library type fr-firststrand. Gene expression values (FPKM = fragments per kilobase per million reads) were computed by Cufflinks v2.2.1 [14] and further normalization between samples was performed using quantile normalization (R/Bioconductor package limma) [15]. Gene normalization and differentially expressed gene (DEG) analysis was performed using the DESeq2 package. A false discovery rate (FDR) of 0.1 was used to correct for multiple testing.

Ingenuity® Pathway Analysis (IPA; Ingenuity® Systems, http://www.ingenuity.com/) was used to search the relevant molecular functions, cellular processes, and canonical pathways [16]. Differentially expressed gene lists were mapped to their corresponding gene objects in the Ingenuity® pathway knowledge base. These so-called focus genes were then used as a starting point for generating biological networks. A score was assigned to each network in the dataset to estimate the relevance of the network to the uploaded gene list. This score reflects the negative logarithm of the *P* value indicating the likelihood of the focus genes being randomly found together in a network. Scores ≥ 2 were considered to be significant with a 99% confidence level. A right-tailed Fisher’s exact test was used to calculate a *p* value determining the probability that the biological function (or pathway) assigned to the data set could be explained by chance alone.

### Real-time quantitative reverse transcription polymerase chain reaction and digital droplet PCR

The conditions of total RNA extraction, complementary DNA synthesis and qRT-PCR were as described previously, and the theoretical basis for N-fold differences in target gene expressions relative to the TBP gene has been described in detail elsewhere [17]. Primers for housekeeping gene *TBP* and the four genes of interest (*CHGB*, *TPTE*, *NMBR*, *KCNK3*) were chosen by using the Oligo6.0 computer program (National Biosciences, Plymouth, MN), and the primers of four genes tested in this study are available on request. Digital droplet PCR (ddPCR) was performed for *ESR1* mutation in pretreatment biopsies according to the protocol described in a previous study and the ddPCR primer design is available on request [18].

### Targeted NGS and somatic mutation data collection

Targeted next-generation sequencing (NGS) was applied to a custom-made panel of 91 “breast cancer-specific” genes selected for their involvement in breast cancer. This BreastCurie panel is composed of the most frequently mutated genes (mutation frequency higher than 1%) in breast cancer from the cancer genome atlas (TCGA) [19] and genes with potential targetable mutations. The list of genes and group of pathways are presented in Additional file 1: Table S1.

DNA was extracted from formalin-fixed paraffin-embedded tissues with the NucleoSpin tissue kit (Macherey-Nagel), and 50 ng of DNA was used for NGS. Targeted sequencing was performed using Illumina Hiseq2500 technology according to the manufacturer’s instructions (Illumina, San Diego, CA, USA). Sequence data were aligned to the human reference genome (hg19) using Bowtie2 algorithm. Median depth was 607× and 87% of targets achieved 5% 100× depth. Single-nucleotide variants (SNVs) and indels were called using GATK UnifiedGenotyper with default parameters. Genes were classified as oncogenes or tumor suppressor genes based on the literature. The Catalog of Somatic Mutations in Cancer (COSMIC) was used to confirm non-synonymous, exonic/splice variants observed at a frequency lower than 0.1% in the population. Moreover, non-COSMIC frameshifts, splice-site, and stop-gain variants were added for tumor suppressor genes. Further confirmation of detected variants was performed by comparison with public databases (cancerhotspot, cbioportal, tumorportal, oncoKB). Detected mutations were then classified as pathogenic variants, unknown pathogenic variants, and non-pathogenic variants.

### Statistical analysis

Statistical analysis was performed with GraphPad Prism (version 5.01) software. Results were considered to be statistically significant at a *p* value < 0.05 (*), < 0.01 (**), or < 0.001 (***).Wilcoxon’s paired test was used to compare pre-NET and post-NET levels of TILs and Mann-Whitney test was used to compare pre-NET and post-NET levels of TILs between responders and non-responders. Contingency tables were constructed and Fisher’s exact tests and Mann-Whitney tests were performed to compare gene mutation profiles between responders and non-responders. Follow-up was measured from the date of randomization to the date of last news for patients with no events. Relapse-free survival (RFS) was determined as the interval between initial diagnosis and detection of the first relapse regardless of its site (local, regional, or distant) and death from any cause. Survival distributions were estimated by the Kaplan-Meier method, and survival was compared between groups with the log-rank test. *P* values were based on the Wald test, and patients with one or more missing data were excluded. All statistical tests were two-sided at the 5% level of significance.

## Results

**Fig. 1:**
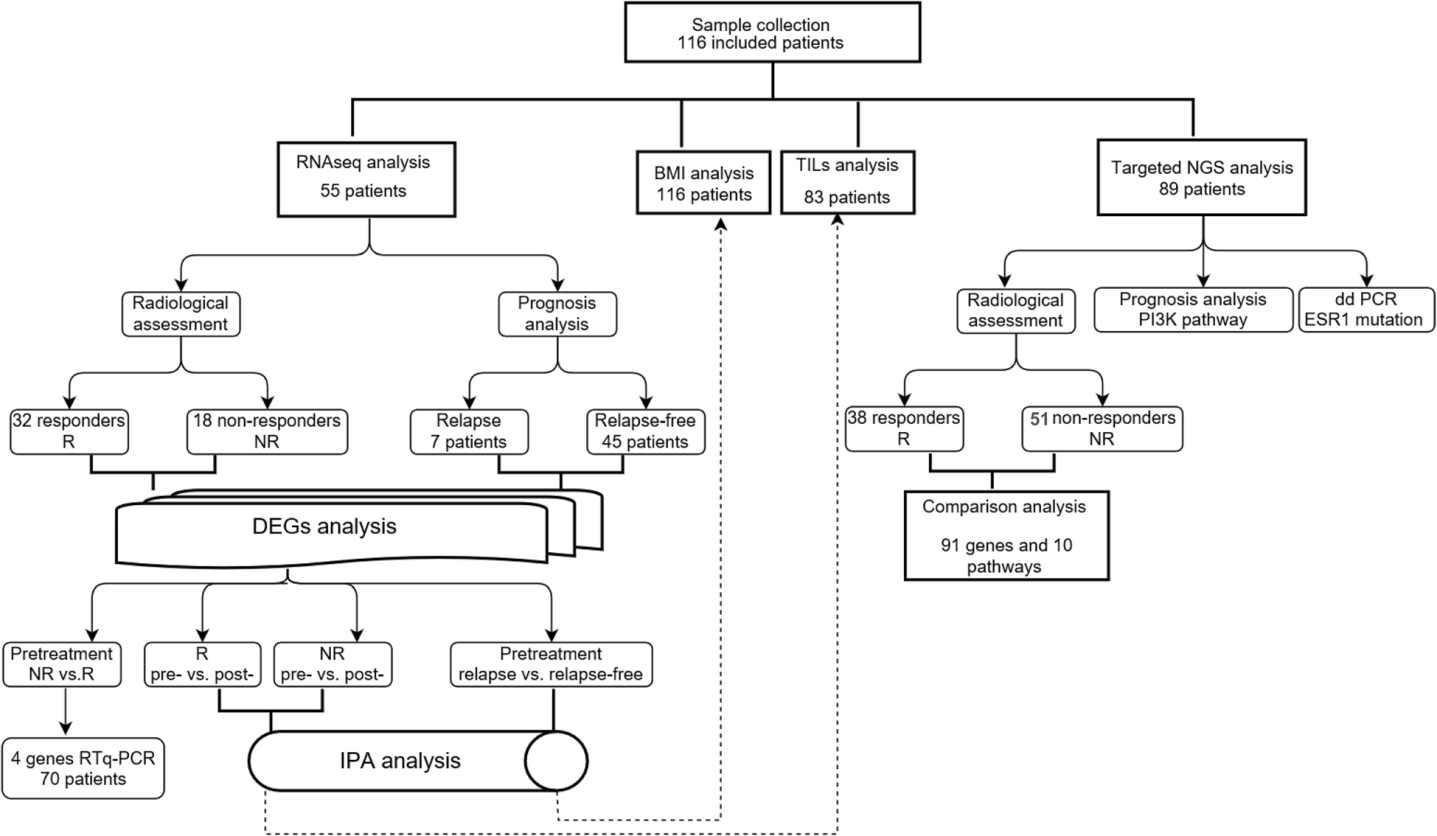
Flow-chart of data processing and analysis. *BMI* body mass index

A study flow chart with methods used for analysis is shown in Fig. 1, and the graphic table of contents is shown in Additional file 2: Figure S1.

### Clinicopathological and molecular response

In the set of 55 tumors with RNA sequencing data, 26 patients had received anastrozole and 29 patients had received fulvestrant. In view of the size of the cohort and the similarities of the transcriptional response to anastrozole and fulvestrant observed in a previous study [20], data from the two arms were pooled for analysis. The baseline characteristics of the 55 patients are summarized in Table 1. The clinical response rate was 61.8% (34 clinical responders, 21 clinical non-responders). The pathological response rate was 16.4% (9 pathological responders and 46 pathological non-responders). Radiological assessment was available for 50 tumors, and objective response rates (complete and partial responses) were 64% (32 responders and 18 non-responders). Clinical, pathological, and radiological responses were used as various methods of evaluation for DEG analysis. The DEGs in pre- and post-NET samples from responders evaluated by these various methods are shown in Additional file 3: Figure S2. As clinical response is quite subjective and as pathological responses were rarely observed, leading to unbalanced groups, radiological response was selected for the following supervised analysis of differentially expressed genes.

**Table 1:** Baseline characteristics of the patients whose samples were analyzed by RNAseq (*n* = 55)

	Number of samples (%)
Anastrozole therapy (Arm A)	26 (47.3)
Fulvestrant therapy (Arm B)	29 (52.7)
Age, mean (range), years	72.1 (51–88)
Age	
≤ 70 years	21 (38.2)
> 70 years	32 (61.8)
ECOG PS	
0	45 (81.8)
1	10 (18.2)
Hormone replacement therapy	
Yes	18 (32.7)
No	37 (67.2)
Tumor staging	
T2	45 (81.8)
T3	7 (12.7)
T4	3 (5.5)
Node staging	
N0	35 (63.6)
N1	17 (30.9)
N2	2 (3.6)
N3	1 (1.7)
Elston-Ellis grade	
I	11 (20.0)
II	38 (69.1)
III	6 (10.9)
Histological type	
Ductal	37 (67.3)
Lobular	15 (27.3)
Other	3 (5.4)
Allred score: ER	
5	1 (1.8)
6	3 (5.5)
7	7 (12.7)
8	44 (80.0)
Allred score: PR	
0–5	21 (38.2)
6	11 (20.0)
7	11 (20.0)
8	12 (21.8)
Ki-67	
≥ 20%	14 (25.5)
**<** 20%	39 (70.9)
ND	2 (3.6)

*ECOG* Eastern Cooperative Oncology Group, *ER* estrogen receptor, *ND* not determined, *PR* progesterone receptor, *PS* performance status

### DEGs in pretreatment tumors between non-responders and responders

Pretreatment samples from 32 responders were first compared to those of 18 non-responders: 30 genes were found to be significantly differentially expressed (Table 2). Twenty-three of these 30 DEGs were relatively overexpressed and 7 genes were relatively underexpressed in responding tumors. Tyrosine-sulfated secretory protein encoding gene *CHGB*, PTEN-related encoding gene *TPTE*, and G protein-coupled receptor encoding gene *NMBR* were significantly overexpressed in responders (FDR < 0.05). Intriguingly, *CHGB*, *TPTE* were upregulated after months of NET in responders, but not in non-responders. In contrast, potassium channel proteins encoding gene *KCNK3* was overexpressed and upregulated after NET in non-responders.

**Table 2:** Genes differentially expressed between non-responders and responders in pretreatment samples

Gene symbol	Description	log2 FC (R/NR)
Upregulated genes		
*CHGB*	Chromogranin B	1.3
*TPTE*	Transmembrane phosphatase with tensin homology	1.2
*NMBR*	Neuromedin-B-preferring bombesin receptor	1.0
*MYO3B*	Myosin IIIB	1.1
*CEP72*	Centrosomal protein 72	0.9
*GRB14*	Growth factor receptor bound protein 14	1.0
*MEGF10*	Multiple epidermal growth factor-like domains protein 10	1.0
*RP5*-*1043L13*,*1*	LncRNA	1.0
*ZXDC*	ZXD family zinc finger C	0.3
*VSX1*	Visual system homeobox 1	0.9
*MBTPS1*	Membrane bound transcription factor peptidase, site 1	0.6
*CTRL*	Chymotrypsin Like	0.4
*SESN3*	Sestrin 3	0.9
*PSKH1*	Protein serine kinase H1	0.4
*TPPP*	Tubulin polymerization promoting protein	1.0
*LINC00578*	Long intergenic non-protein coding RNA 578	1.0
*RP11*-*712B9*,*2*	LncRNA	0.9
*GBA3*	Glucosylceramidase beta 3	0.9
*CPLX2*	Complexin 2	0.7
*HSDL1*	Hydroxysteroid dehydrogenase like 1	0.5
*AC008174*,*3*	LncRNA	0.9
*FUK*	Fucokinase	0.5
*MT1L*	Metallothionein 1L	0.9
Downregulated genes		
*ASAH1*	N-Acylsphingosine amidohydrolase 1	− 0.8
*ANK1*	Ankyrin 1	− 1.0
*PCSK1*	Proprotein convertase subtilisin/kexin type 1	− 0.9
*SEZ6L*	Seizure related 6 homolog like	− 0.9
*TMC3*	Transmembrane channel like 3	− 0.9
*RP11*-*761I4*,*3*	LncRNA	− 1.0
*KCNK3*	Potassium two pore domain channel subfamily K member 3	− 0.9

Genes were selected by class comparison analysis at a FDR of 0.1; *FC* fold change, *NR* non-responder, *R* responder

### qRT-PCR results

Four of these genes (*CHGB*, *TPTE*, *NMBR*, *KCNK3*) were tested by qRT-PCR in 70 RNA pretreatment samples from CARMINA02 patients. *NMBR* and *KCNK3* expression changes were in accordance with RNA sequencing analysis results, although the differences between responders and non-responders were not statistically significant (data not shown). As *CHGB* and *TPTE* expression were detected on few samples, further analysis was not performed.

### Differences of transcriptional response to NET between responders and non-responders

**Fig. 2:**
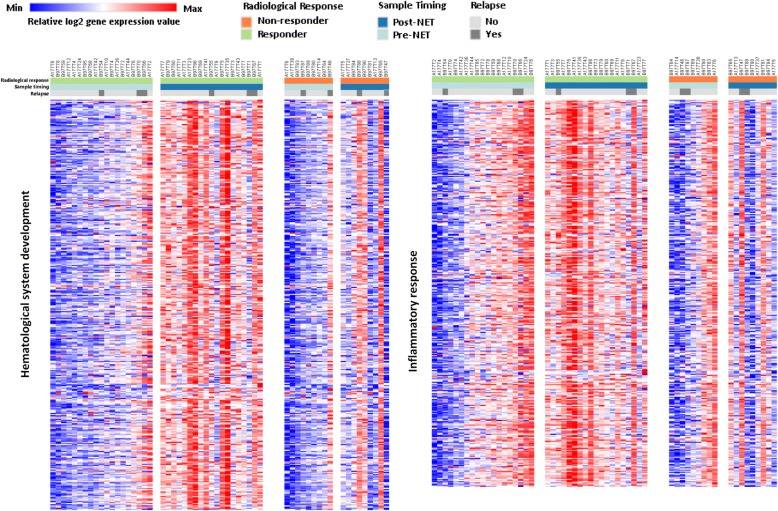
Heatmaps summarizing changes in gene expression over time in two major molecular functional groups in NET responders and non-responders. Samples are ordered from left to right by patient identification for each time point. Red and blue represent relative high and low log2 gene expression values, respectively

**Fig. 3:**
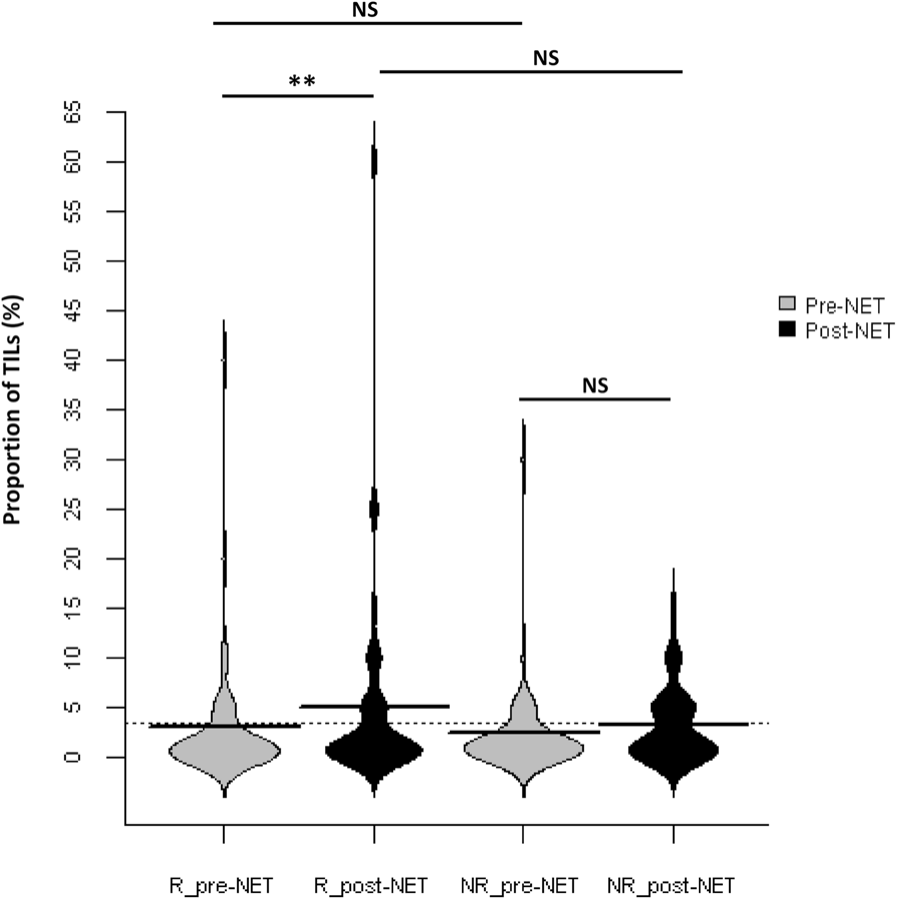
Beanplot summarizing the changes in TILs before and after NET in responders and non-responders. *R*_*pre*-*NET* pre-neoadjuvant endocrine therapy samples in responders, *R*_*post*-*NET* post-neoadjuvant endocrine therapy samples in responders, *NR*_*pre*-*NET* pre-neoadjuvant endocrine therapy samples in non-responders, *NR*_*post*-*NET* post-neoadjuvant endocrine therapy samples in non-responders. *NS* not significant. **p value < 0.01

Gene expression data were available pretreatment and after 6 months NET for 32 responders (19 paired samples and 13 pretreatment-only samples) and 18 non-responders (9 paired samples and 9 pretreatment-only samples). Using multiple testing corrected class comparison analysis, 1085 genes were significantly differentially expressed at a FDR of 0.1 in 32 responders: 707 of these genes were upregulated and 378 were downregulated, and 138 genes were significantly differentially expressed in 18 non-responders: 28 genes were upregulated and 110 genes were downregulated (Additional file 4: Table S2). Ingenuity® pathway analysis showed that immune-related genes and inflammatory response-associated genes were predominantly observed in responders. The top ten statistically significantly altered pathways are presented in Table 3. Immune molecule-related pathways, such as *Th1 pathway*, *role of NFAT in regulation of the immune response* pathway, were significantly activated. IPA upstream regulator analysis showed that pro-inflammatory cytokines IL2, lipopolysaccharide, and IL12 (complex) were the top 3 activated molecules. IPA molecular function exploration showed that hematological system development and immune-inflammatory response were significantly upregulated by NET in responding tumors. However, all these significant transcriptional responses to NET observed in responders were not detected in non-responders, but remained relatively unchanged. To visualize the degree of variability of the transcriptional response to NET between responders and non-responders, hematological system development and inflammatory response-related genes were clustered in heat maps for 28 paired pretreatment and post-treatment tumors (Fig. 2). Moreover, cell cycle and proliferation-related pathways, *mitotic roles of polo*-*like kinase*, *cyclins and cell cycle regulation*, and *p53 signaling* pathways were inhibited in responders after NET. In tumors from non-responders (*n* = 18), *mitotic roles of polo*-*like kinase*, *cyclins and cell cycle*, and *cell cycle*: *G2*/*M DNA damage checkpoint regulation* were the top 3 regulated pathways but none of these pathways was significantly altered due to the small number of genes affected. Cell cycle and proliferation-associated genes were downregulated in non-responders, but with a lower “inhibited” prediction scores than in responders. Comparative analysis of disease and biological function by IPA showed that lymphocytes, mainly T lymphocyte, presented activated function in responders, but inhibited function in non-responders. Following the observation of an association between local immune system activation and response, TILs were assessed in 43 and 40 paired pretreatment and post-treatment samples from responders and non-responders, respectively. The percentage of post-NET TILs was significantly increased compared to pre-NET TILs in responders (mean% 5.07 ± 10.42 vs. 3.047 ± 6.859, *p* = 0.0071), but not in non-responders (mean% 3.15 ± 3.648 vs. 2.425 ± 4.919, *p* = 0.0938). No significant difference in terms of pre-NET TILs or post-NET TILs was observed between responders and non-responders (*p* = 0.3862, *p* = 0.8672, respectively) (Fig. 3).

**Table 3:** Top 10 statistically significantly altered pathways in samples from responders to NET

Ingenuity canonical pathways	-log (*p* value)	Number of genes and overlapping ratio (%)	z-score
Th1 Pathway	22.3	40 (29.6)	4.7
iCOS-iCOSL signaling in T helper cells	17.0	33 (26.8)	4.3
CD28 signaling in T helper cells	16.9	34 (25.8)	3.8
PKCθ signaling in T lymphocytes	14.2	31 (23.3)	4.4
Role of NFAT in regulation of the immune response	12.9	34 (18.8)	5.0
B Cell receptor signaling	7.9	28 (14.7)	3.5
Calcium-induced T lymphocyte apoptosis	7.9	16 (24.2)	4.0
Tec kinase signaling	7.8	26 (15.3)	3.7
PI3K signaling in B lymphocytes	7.5	22 (16.9)	4.1
Neuroinflammation signaling pathway	6.7	35 (11.3)	2.7

Only top canonical pathways with predictive z-score ≥ 2 are presented, together with a *p* value. The overlapping ratio indicates the ratio of genes from the dataset that map to the pathway divided by the total number of genes that map to the same pathway

### Differences of transcriptional expression according to prognosis

**Fig. 4:**
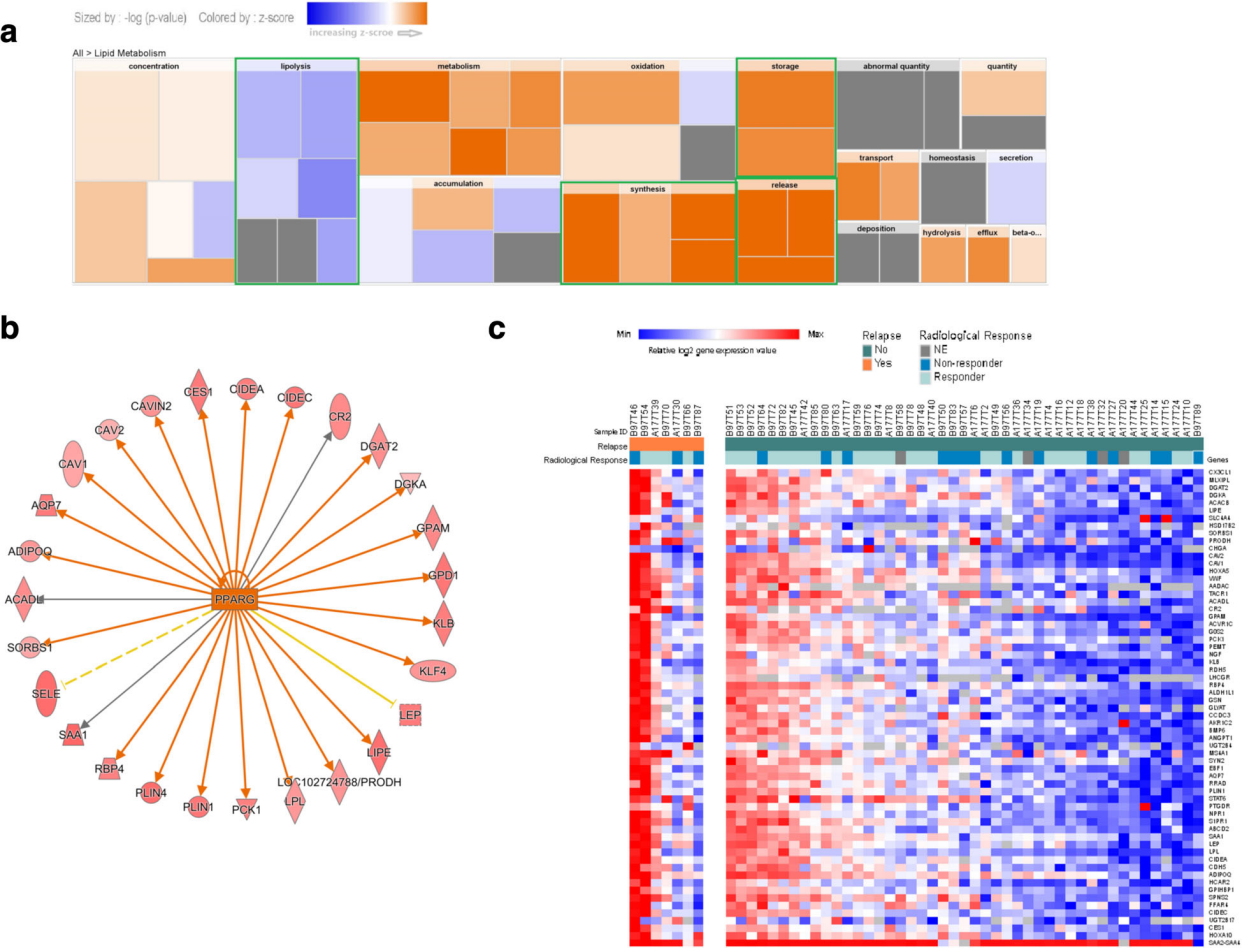
Comparative analysis of lipid metabolism between 7 tumors with relapse and 45 tumors without relapse. **a** The IPA disease/function analysis confirmed that gene level alterations corresponded to lipid metabolism. The calculated z-score indicates a bio-function with genes exhibiting overall increased mRNA levels (orange bars) or decreased mRNA levels (blue bars). The most significantly altered bio-function bars are highlighted with a green border. **b** Upstream regulator analysis of PPARγ in lipid metabolism. Upregulated genes are highlighted in red and color depth is correlated with fold change. Orange lines with arrows indicate direct activation. Solid and dashed lines represent direct and indirect interactions, respectively. Yellow and gray lines depict inconsistent effects and no prediction, respectively. **c** Heatmap summarizing the changes in lipid metabolism-related gene expression in tumors with and without relapse. Red and blue represent relative high and low log2 gene expression values, respectively. *NE* not evaluated

RNAseq gene expression data of 7 tumors from patients who relapsed were compared to those of 45 tumors from relapse-free patients. A total of 273 genes were differentially expressed with 246 genes upregulated and 27 genes downregulated (Additional file 5: Table S3). IPA analysis of differentially expressed genes showed that lipid metabolism molecular function was significantly different. In the lipid metabolism process, synthesis, storage, and release functions were activated, but the lipolysis function was inhibited in tumors with relapse (Fig. 4a). The lipid metabolism-related pathway LXR/RXR activation was activated and FXR/RXR activation and triacylglycerol degradation pathways were also identified as two of the top three pathways, but with no prediction score according to IPA analysis. Peroxisome proliferator activated receptor gamma (PPARγ) was predicted to be the most intensely activated upstream regulator (-log_10_ *p* value = 11.4) (Fig. 4b). The expression heatmaps of 64 genes from 52 tumors with enriched lipid metabolism function are grouped and shown in Fig. 4c. In the light of these data, body mass index (BMI) data from patients in the CARMINA02 trial were reviewed, but no significant difference was observed between patients with and without relapse (26.9 ± 5.2 vs. 26.1 ± 4.5, respectively, *p* = 0.5).

### Somatic mutation profiling

**Fig. 5:**
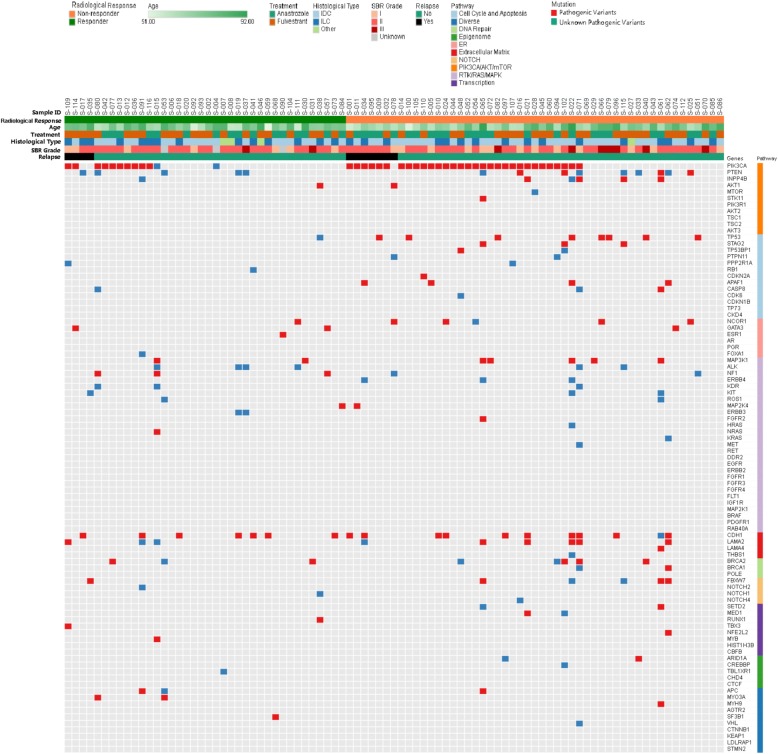
Deleterious somatic mutations in 89 tumor samples. Tumors with available mutation data are grouped by radiological response along the *x*-axis, also showing clinicopathological characteristics for each tumor on the *x*-axis, the 91 genes of BreastCurie panel are enriched to 10 signaling on the *y*-axis. The somatic mutations of each tumor are indicated by colored boxes; red boxes indicate pathogenic variants, and blue boxes indicate unknown pathogenic variants

**Fig. 6:**
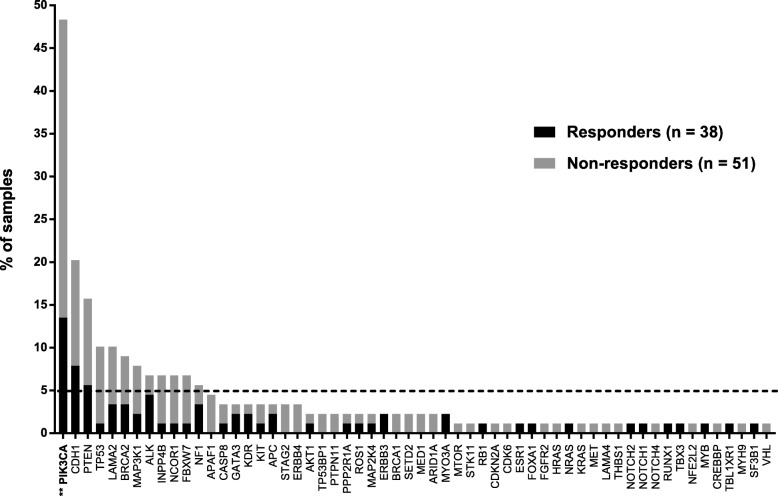
Somatic mutation frequency and comparison of somatic mutation frequency between responders and non-responders. Data show the percentage of samples with somatic mutations on our 91-gene panel; gray bars indicate non-responders, black bars indicate responders. **p value < 0.01, comparison of PIK3CA mutation frequency between responders and non-responders

The baseline characteristics and relation with radiological response for the 89 patients analyzed by targeted NGS are summarized in Table 4. Analysis of the 91 genes sequenced from 89 samples revealed 243 somatic mutations, comprising 225 point mutations and 18 indels. Point mutations included 146 missense, 58 nonsense, and 21 splice-site mutations and 18 indels included 16 frame-shift and 2 in-frame mutations. These mutations corresponded to 181 different mutations on 58 genes. Seventy-four samples exhibited at least one mutation. After annotation of these mutations, 243 mutations were classified as pathogenic variants and unknown pathogenic variants (Fig. 5). All pathogenic variants and unknown pathogenic variants were selected for further analysis. The most frequently mutated genes (≥ 5.0%) in this cohort were *PIK3CA* (48.3%), *CDH1* (20.2%), *PTEN* (15.7%), *TP53* (10.1%), *LAMA2* (10.1%), *BRCA2* (9.0%), *MAP3K1* (7.9%), *ALK* (6.7%), *INPP4B* (6.7%), *NCOR1* (6.7%), and *NF1* (5.6%) (Fig. 6).

**Table 4:** Baseline characteristics and relation with radiological response of the patients whose samples were analyzed by targeted NGS (*n* = 89)

	Total No. (%)	Responders No. (%)	Non-responders No. (%)	*p*
Treatment				NS
Anastrozole (Arm A)	41 (46.1)	16 (42.1)	25 (49.0)	
Fulvestrant (Arm B)	48 (53.9)	22 (57.9)	26 (51.0)	
Age, mean (range), years	71.9 (51–92)	72.1 (51–87)	72.0 (53–92)	NS
Age				NS
≤ 70 years	41 (46.1)	14 (36.8)	27 (52.9)	
> 70 years	48 (53.9)	24 (63.2)	24 (41.1)	
ECOG PS				NS
0	75 (84.3)	33 (86.8)	42 (82.4)	
1	14 (15.7)	5 (13.2)	9 (17.6)	
Hormone replacement therapy				NS
Yes	30 (33.7)	15 (39.5)	15 (29.4)	
No	59 (66.3)	24 (60.5)	35(68.6)	
Tumor staging				NS
T2	74 (83.1)	31 (81.6)	43 (84.3)	
T3	11 (12.4)	4 (10.5)	7 (13.7)	
T4	4 (4.5)	3 (7.9)	1 (2.0)	
Node staging				NS
N0	66 (63.6)	24 (63.2)	42 (82.4)	
N1	21 (30.9)	13 (34.2)	8 (15.7)	
N2	2 (3.6)	1 (2.6)	1 (2.0)	
N3	0 (1.7)	0 (0.0)	0 (0.0)	
Elston-Ellis grade				NS
I	20 (22.5)	7 (18.4)	13 (25.5)	
II	57 (64.0)	26 (68.4)	31 (60.8)	
III	9 (10.1)	2 (5.3)	7 (13.7)	
Unknown	3 (3.4)	3(7.9)	0(0.0)	
Histological type				NS
Ductal	55 (61.8)	21 (55.3)	34 (66.7)	
Lobular	30 (33.7)	14 (36.8)	16 (31.4)	
Other	4 (4.5)	3 (7.9)	1 (1.9)	
Allred score: ER				NS
4–5	2 (2.2)	2 (5.3)	0 (0.0)	
6	4 (4.5)	3 (7.9)	1 (2.0)	
7	13 (14.6)	5 (13.1)	8 (15.7)	
8	70 (78.7)	28 (73.7)	42 (82.3)	
Allred score: PR				NS
0–5	38 (42.7)	14 (36.8)	24 (47.1)	
6	14 (15.7)	9 (23.7)	5 (9.8)	
7	20 (22.5)	7 (18.4)	13 (25.5)	
8	17 (19.1)	8 (21.1)	9 (17.6)	
Ki-67				NS
≥ 20%	25 (28.1)	9 (23.7)	16 (31.4)	
** <** 20%	61 (68.5)	28 (73.7)	33 (64.7)	
ND	3 (3.4)	1 (2.6)	2 (3.9)	

*ECOG* Eastern Cooperative Oncology Group, *ER* estrogen receptor, *ND* not determined, *PR* progesterone receptor, *PS* performance status, *NS* no significance

### Comparison of somatic mutations between responders and non-responders

**Fig. 7:**
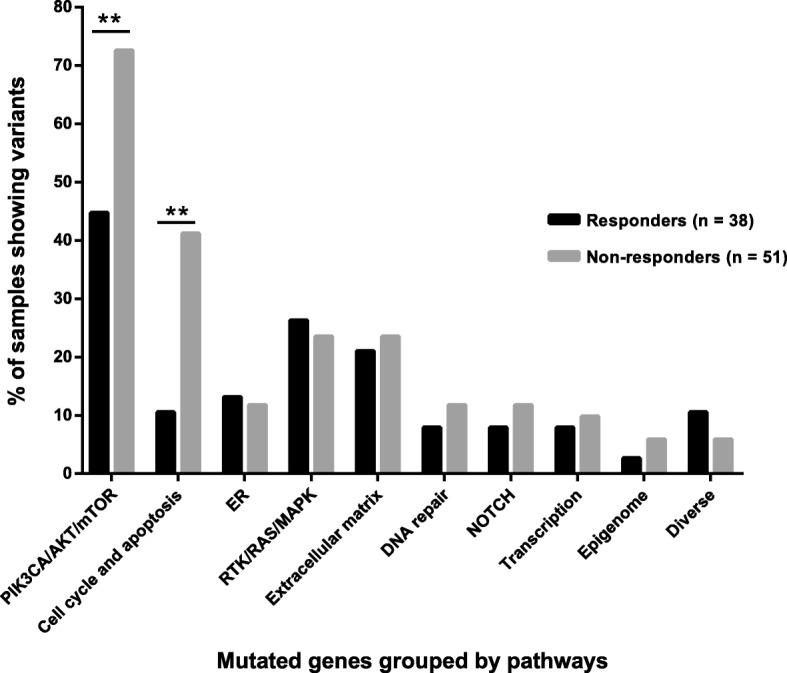
Comparison of somatic mutation frequency grouped by biological pathways in responders and non-responders. Data show the percentage of samples with alteration on ten biological pathways; gray bars indicate non-responders, black bars indicate responders. **p value < 0.01 (**)

The average number of mutations per sample was higher in non-responders (2.88 vs. 1.64, *p =* 0.03) with a different mutation frequency between responders and non-responders. *PIK3CA* was mutated significantly more frequently in non-responders (60.8 vs. 31.6% *p* = 0.0098) (Fig. 6). For greater clarity, the 91 genes were grouped into ten different signaling pathways and the pathway was considered to be altered when at least one gene of the pathway was mutated. The frequency of alteration of the cell cycle and apoptosis pathway and PIK3CA/AKT/mTOR pathway was significantly different between responders and non-responders (10.5 vs. 41.2%, *p* = 0.0017, 44.7 vs. 72.5%, *p* = 0.0094, respectively) (Fig. 7).

### Comparison of somatic mutations according to prognosis

**Fig. 8:**
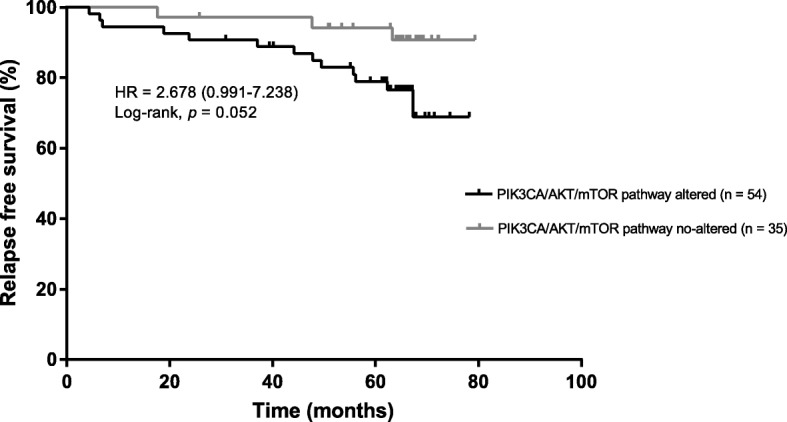
RFS according to PIK3CA/AKT/mTOR pathway status. Kaplan-Meier estimates of RFS according to PIK3CA/AKT/mTOR pathway status in patients; 54 patients with at least 1 mutation in the PIK3CA/AKT/mTOR pathway, 35 patients with no mutation in the PIK3CA/AKT/mTOR pathway

The average number of mutations per sample was not significantly different between tumors with relapse and tumors without relapse (2.7 vs. 2.6, *p =* 0.9) and the mutation frequency was similar in the two groups (data not shown). After grouping into pathways, the PIK3CA/AKT/mTOR pathway was altered significantly more frequently in tumors with relapse (90.9 vs. 56.4%, *p* = 0.004). The association between PIK3CA/AKT/mTOR pathway status and RFS was assessed in 89 patients with a median follow-up of 65.3 months (range 56.0 to 62.6 months). Patients with alteration of the PIK3CA/AKT/mTOR pathway tended to have a poorer RFS than patients with no mutations in this pathway (hazard ratio = 2.7, 95% CI 0.991–7.238, *p* = 0.052) (Fig. 8).

### *ESR1*-activating mutations in primary tumors

To explore *ESR1* mutations related to endocrine resistance in more detail, the sequencing depth filter was decreased to 1%. Targeted NGS analysis showed that seven post-NET samples harbored hotspot mutations on the ligand-binding domain (LBD) of *ESR1*. ddPCR, a more sensitive method, was used to detect *ESR1* mutations on pre-NET samples of these seven patients: three samples (3.4%, 3/89) were mutated and four samples were not informative due to insufficient DNA.

## Discussion

Neoadjuvant endocrine therapy provides an opportunity to investigate treatment-induced molecular changes in order to elucidate sensitivity to treatment. In the present study, we analyzed the transcriptional response induced by NET in a prospective trial on a homogenous population of mainly low-proliferative HR+/HER2− breast cancer. Pretreatment transcriptional profiling differences between NET responders and non-responders were initially analyzed. Unfortunately, transcriptomic analysis revealed few differentially expressed genes and Ingenuity® pathway analysis did not identify any clear signatures able to distinguish endocrine-sensitive tumors from endocrine-resistant tumors. However, a G protein-coupled receptor encoding gene, *NMBR,* is known to play a critical role in tumor development, invasion, and metastasis [21, 22]. In our study, *NMBR* was overexpressed in endocrine-responding tumors, consistent with the results of recent studies on *GRPR*, the most important paralog of *NMBR*. High *GRPR* expression has been recently associated with a lower risk of distant metastases in breast cancer and prolonged progression-free survival after initiation of first-line tamoxifen therapy [23, 24]. In contrast, *KCNK3* was overexpressed and upregulated after NET in non-responders. A low level of *KCNK3* has been found in MCF-7 and MDA-MB-231 breast cancer cells, but the role of *KCNK3* in breast cancer remains unknown at the present time. However, *KCNK3* has been reported to regulate apoptosis and proliferation in lung cancer, and *KCNK3* knockdown enhances apoptosis of tumor cell lines [25]. *NMBR* and *KCNK3* genes may play roles in activities such as cell motility, activation, and proliferation. As the expression levels of these two genes between endocrine-responding tumors and non-responding tumors were not significantly different on qRT-PCR analysis, further research is needed to investigate their predictive value in breast cancer.

A separate, comparative analysis of NET responders and non-responders was carried out to determine whether endocrine therapy affected gene expression and to elucidate the mechanism of sensitivity to endocrine therapy. Many more genes were affected by treatment in responders compared to non-responders. No gene was modulated in opposite directions in these two different response groups. Hematological system and immune response were activated and the Th1 pathway was activated with numerous upregulated genes by endocrine therapy in responding tumors. These observations were consistent with previously published NET studies, demonstrating that neoadjuvant anastrozole and letrozole treatment induce immune response after 2 weeks or 3 months in endocrine-sensitive tumors [5, 10]. Moreover, tumor-infiltrating lymphocyte levels were increased in treatment-sensitive tumors after NET, but remained numerically stable in treatment-insensitive tumors. However, neither pretreatment nor post-treatment levels of TILs were directly associated with objective response to NET. TILs have been reported to be a favorable prognostic factor and possible a predictive factor, particularly in TNBC and HER2-positive breast cancer [26–29], but not in the HR+HER2− subtype. The interaction between tumor cells and the immune milieu appears to involve different mechanisms in HR+HER2− BC compared to other subtypes [30] and further research in this subtype is urgently needed.

Several previous studies have reported that cell cycle- and proliferation-related genes were significantly inhibited in endocrine-responding tumors, but less markedly inhibited or not inhibited in non-responding tumors [4, 10, 20, 31]. In our previous study [11], a significant reduction of Ki-67 level between baseline and post-treatment samples was observed. In the present study, the RNAseq analysis showed that cell cycle- and proliferation-related genes were downregulated in both responders and non-responders. However, these genes were not here the most regulated genes in endocrine-sensitive tumors. Unlike the studies mentioned above [4, 20, 31], cell cycle and proliferation pathway was minimally affected by endocrine therapy in responding tumors and a possible explanation is that more than 70% of tumors in the CARMINA02 cohort are luminal A subtype with low baseline Ki67. To note, post-treatment samples were obtained after 6 months treatment which differ with samples analyzed after 2 or 4 weeks treatment in previous studies. The genes we have identified as significantly regulated after long-term NET may be distinct genes, unrelated with the proliferation pathway regulated after short-term NET. Due to the small number of non-responding tumors analyzed by RNAseq, with a majority of those tumors evaluated as stable disease, few DEGs were detected in non-responding tumors before and after NET, preventing identification of a potential resistance mechanism. However, an inactive immune system may play a role in resistance to endocrine therapy.

RNA analysis showed that lipid metabolism was a key molecular functional mechanism related to prognosis. Activated lipid synthesis and storage, and inhibited lipolysis function were clearly correlated with poorer prognosis, consistent with the results of a previous study showing that dysfunctional lipid metabolism promotes breast cancer [32]. PPARγ with retinoid X receptors (RXRs) promotes differentiation of adipocyte progenitors and preadipocytes in adipose tissue and regulates lipid biosynthesis and storage [33]. PPARγ, the most important upstream regulator, was predicted to be activated: this result suggests that PPARγ could be explored as potential therapeutic target for HR+HER2− breast cancer. Further research to investigate the major factors in lipid metabolism and interventional research of targeting PPARγ in breast cancer are needed.

A cohort of 89 tumors was analyzed for the detection of somatic mutations based on the 91 genes of the custom-made BreastCurie panel. The mutation count was higher in tumors from non-responders, which could constitute a hallmark of increased genomic instability correlated with unresponsiveness to endocrine therapy. However, no significant difference in mutation loads was observed between poor and good prognosis tumors. *PIK3CA* is the gene most frequently mutated in luminal breast cancer and whether these mutations cause resistance to endocrine therapy is a crucial issue. Targeted NGS analysis of tumor DNA demonstrated significantly more frequent PIK3CA mutations and PI3K/AKT/mTOR pathway alterations in non-responders. These results are consistent with those of previous studies [34, 35], as a negative interaction between *PIK3CA* mutation and clinical response to NET was observed in a pooled study comprising 235 patients. PI3K pathway activation has been associated with de novo and acquired resistance to endocrine therapy in in vitro studies [36, 37], and PIK3CA inhibitors have been investigated in clinical trials to reverse endocrine resistance. Encouraging results have been obtained in carefully selected populations and further trials with better-tolerated second-generation PI3K inhibitors are ongoing [38]. Eight samples with *TP53* stop-gain mutations were detected among the non-responding tumors, in contrast with only one *TP53* mutation in responding tumors. *TP53* mutation has been reported to be a poor prognostic factor in HR-positive breast cancer and is correlated with endocrine resistance [39–41]. Other cell cycle and apoptosis pathway gene mutations, such as *STAG2*, *APAF1*, *TP53BP1*, and *PTPN11* mutations, were only found in non-responding tumors. No study has yet investigated whether these genes are associated with resistance to endocrine therapy.

*ESR1* mutation drives acquired endocrine resistance. *ESR1* mutation has been studied in metastatic breast cancer, as *ESR1* mutations are a very rare event in primary breast cancers with only 0.5% in a large TCGA dataset [42–44]. However, the rare mutant ESR1 LBD cells may arise from a rare and undetectable pre-existing clone in primary treatment-naive tumors, conferring a selective advantage over other endocrine-sensitive clones in response to endocrine therapy [45]. In our study, *ESR1* hotspot mutations were detected in 3.4% of treatment-naive tumors, and interestingly all of these tumors were non-responders to NET. Detection of *ESR1* mutation as early as possible may therefore be useful for prognosis and clinical decision-making, but screening in early-stage disease would likely require very sensitive techniques. Emerging platforms such as digital droplet PCR allow the detection and quantification of these mutations [18, 46].

## Conclusions

Overall, transcriptional response to neoadjuvant endocrine therapy in HR-positive/HER2-negative low-proliferative tumors varies considerably between endocrine responding and non-responding tumors. Th1-related immune system appears to play an important role, as confirmed by increased levels of TILs in response to endocrine therapy in responding tumors. Comparative analyses of pre-/post-NET samples in NET trials are rare and these findings require to be confirmed on a bigger cohort. In terms of survival, lipid metabolism was shown to affect prognosis in this population. Targeted NGS analysis revealed a high frequency of clinically relevant somatic mutations, particularly in PIK3CA/AKT/mTOR and the cell cycle and apoptosis pathway, in endocrine-resistant tumors. Finally, *ESR1* mutations were detected in treatment-naive tumors with sensitive technologies, and could be useful to guide the choice of endocrine therapy.

## Additional files

Additional file 1: **Table S1.** Breast Curie gene panel for targeted NGS. (DOCX 21 kb)

Additional file 2: **Figure S1.** Graphic table of contents. (PDF 561 kb)

Additional file 3: **Figure S2.** Number of overlapping DEGs on pre- and post-NET in responders defined by three different response assessments. Venn diagram showing the total numbers and overlapping numbers of differentially expressed genes pre-NET compared to post-NET based on RNA sequencing data for responding patients using three different evaluation methods. DEGs: differentially expressed genes. (PDF 15 kb)

Additional file 4: **Table S2.** Genes differentially expressed between post- and pre-NET in samples from responders and non-responders. (DOCX 82 kb)

Additional file 5: **Table S3.** Genes differentially expressed between relapsed tumors and non-relapsed tumors. (DOCX 23 kb)

## Abbreviations

BMI: Body mass index
DEGs: Differentially expressed genes
FDR: False discovery rate
FFPE: Formalin-fixed and paraffin-embedded
HER2: Human epidermal growth factor receptor 2
HR: Hormone receptor
IHC: Immunohistochemistry
IPA: Ingenuity® pathway analysis
LBD: Ligand-binding domain
NET: Neoadjuvant endocrine therapy
NGS: Next-generation sequencing
PEPI: Preoperative endocrine prognostic index
PIK3CA: Phosphatidylinositol 3-kinase: catalytic: alpha polypeptide gene
PPARγ: Peroxisome proliferator activated receptor gamma
qRT-PCR: Real-time quantitative reverse transcription polymerase chain reaction
RFS: Relapse-free survival
TCGA: The cancer genome atlas
TILs: Tumor-infiltrating lymphocytes
