# Supplementary material for: Molecular profiling of hormone receptor-positive, HER2-negative breast cancers from patients treated with neoadjuvant endocrine therapy in the CARMINA 02 trial (UCBG-0609)
Source: J Hematol Oncol. 2018 Oct 11;11:124. doi: 10.1186/s13045-018-0670-9 (PMC6180434; doi:10.1186/s13045-018-0670-9)
Supplement: Supplementary file 1 — Table S1. Breast Curie gene panel for targeted NGS. (DOCX 21 kb) [file 13045_2018_670_MOESM1_ESM.docx]

**Table S1. BreastCurie gene panel for targeted NGS**

| **Gene name** | **NM_** | **Pathway** |
| --- | --- | --- |
| *CDKN1B* | 004064.4 | Cell cycle and apoptosis |
| *PPP2R1A* | 014225.5 |  |
| *PTPN11* | 002834.3 |  |
| *RB1* | 000321.2 |  |
| *TP53* | 000546.4 |  |
| *STAG2* | 006603.4 |  |
| *TP53BP1* | 005657.2 |  |
| *CDK4* | 000075.3 |  |
| *CDK6* | 001259.6 |  |
| *TP73* | 005427.3 |  |
| *CDKN2A* | 000077.4 |  |
| *APAF1* | 181861.1 |  |
| *CASP8* | 001228.4 |  |
| *FBXW7* | 033632.3 | NOTCH |
| *NOTCH1* | 017617.3 |  |
| *NOTCH2* | 024408.3 |  |
| *NOTCH4* | 004557.3 |  |
| *ARID1A* | 006015.4 | Epigenome |
| *CTCF* | 006565.3 |  |
| *TBL1XR1* | 024665.4 |  |
| *CREBBP* | 004380.2 |  |
| *CHD4* | 001273.2 |  |
| *ESR1* | 000125.3 | ER |
| *FOXA1* | 004496.3 |  |
| *GATA3* | 002051.2 |  |
| *NCOR1* | 006311.3 |  |
| *PGR* | 000926.4 |  |
| *AR* | 000044.3 |  |
| *BRAF* | 004333.4 | RTK/RAS/MAPK |
| *EGFR* | 005228.3 |  |
| *FGFR1* | 023110.2 |  |
| *FGFR2* | 000141.4 |  |
| *FGFR3* | 000142.4 |  |
| *FGFR4* | 002011.4 |  |
| *ERBB2* | 004448.3 |  |
| *ERBB3* | 001982.3 |  |
| *ERBB4* | 005235.2 |  |
| *HRAS* | 005343.2 |  |
| *IGF1R* | 000875.4 |  |
| *KRAS* | 004985.4 |  |
| *MAP2K1* | 002755.3 |  |
| *MAP2K4* | 003010.3 |  |
| *MAP3K1* | 005921.1 |  |
| **Gene name** | **NM_** | **Pathway** |
| *MET* | 000245.2 | RTK/RAS/MAPK |
| *NF1* | 000267.3 |  |
| *NRAS* | 002524.4 |  |
| *PDGFRA* | 006206.4 |  |
| *RAB40A* | 080879.2 |  |
| *RET* | 020975.4 |  |
| *ROS1* | 002944.2 |  |
| *FLT1* | 002019.4 |  |
| *KDR* | 002253.2 |  |
| *ALK* | 004304.4 |  |
| *DDR2* | 006182.2 |  |
| *KIT* | 000222.2 |  |
| *THBS1* | 003246.2 | Extracellular matrix |
| *CDH1* | 004360.3 |  |
| *LAMA2* | 000426.3 |  |
| *LAMA4* | 002290.4 |  |
| *AKT1* | 005163.2 | PIK3CA/AKT/mTOR |
| *AKT2* | 001626.5 |  |
| *AKT3* | 005465.4 |  |
| *INPP4B* | 003866.2 |  |
| *MTOR* | 004958.3 |  |
| *PIK3CA* | 006218.2 |  |
| *PIK3R1* | 181523.2 |  |
| *PTEN* | 000314.4 |  |
| *STK11* | 000455.4 |  |
| *TSC1* | 000368.4 |  |
| *TSC2* | 000548.3 |  |
| *BRCA1* | 007294.3 | DNA repair |
| *BRCA2* | 000059.3 |  |
| *POLE* | 006231.2 |  |
| *RUNX1* | 001754.4 | Transcription |
| *TBX3* | 016569.3 |  |
| *NFE2L2* | 006164.3 |  |
| *MYB* | 005375.2 |  |
| *HIST1H3B* | 003537.3 |  |
| *SETD2* | 014159.6 |  |
| *MED1* | 004774.3 |  |
| *CBFB* | 001755.2 |  |
| *KEAP1* | 012289.3 | Diverse |
| *LDLRAP1* | 015627.2 |  |
| *STMN2* | 007029.3 |  |
| *MYO3A* | 017433.4 |  |
| CTNNB1 | 001904.3 |  |
| *VHL* | 000551.3 |  |
| *AGTR2* | 000686.4 |  |
| **Gene name** | **NM_** | **Pathway** |
| *APC* | 000038.5 | Diverse |
| *SF3B1* | 012433.2 |  |
| *MYH9* | 002473.4 |  |
