# Supplementary material for: Molecular profiling of hormone receptor-positive, HER2-negative breast cancers from patients treated with neoadjuvant endocrine therapy in the CARMINA 02 trial (UCBG-0609)
Source: J Hematol Oncol. 2018 Oct 11;11:124. doi: 10.1186/s13045-018-0670-9 (PMC6180434; doi:10.1186/s13045-018-0670-9)

We investigated the molecular profiling of HR-positive, HER2-negative breast cancers patients treated with NET, and identified Th1-related immune system and lipid metabolism play roles and targeted NGS analysis revealed a high frequency of clinically relevant somatic mutations, particularly in PIK3CA/AKT/mTOR and the cell cycle and apoptosis pathway in endocrine-resistant tumors.

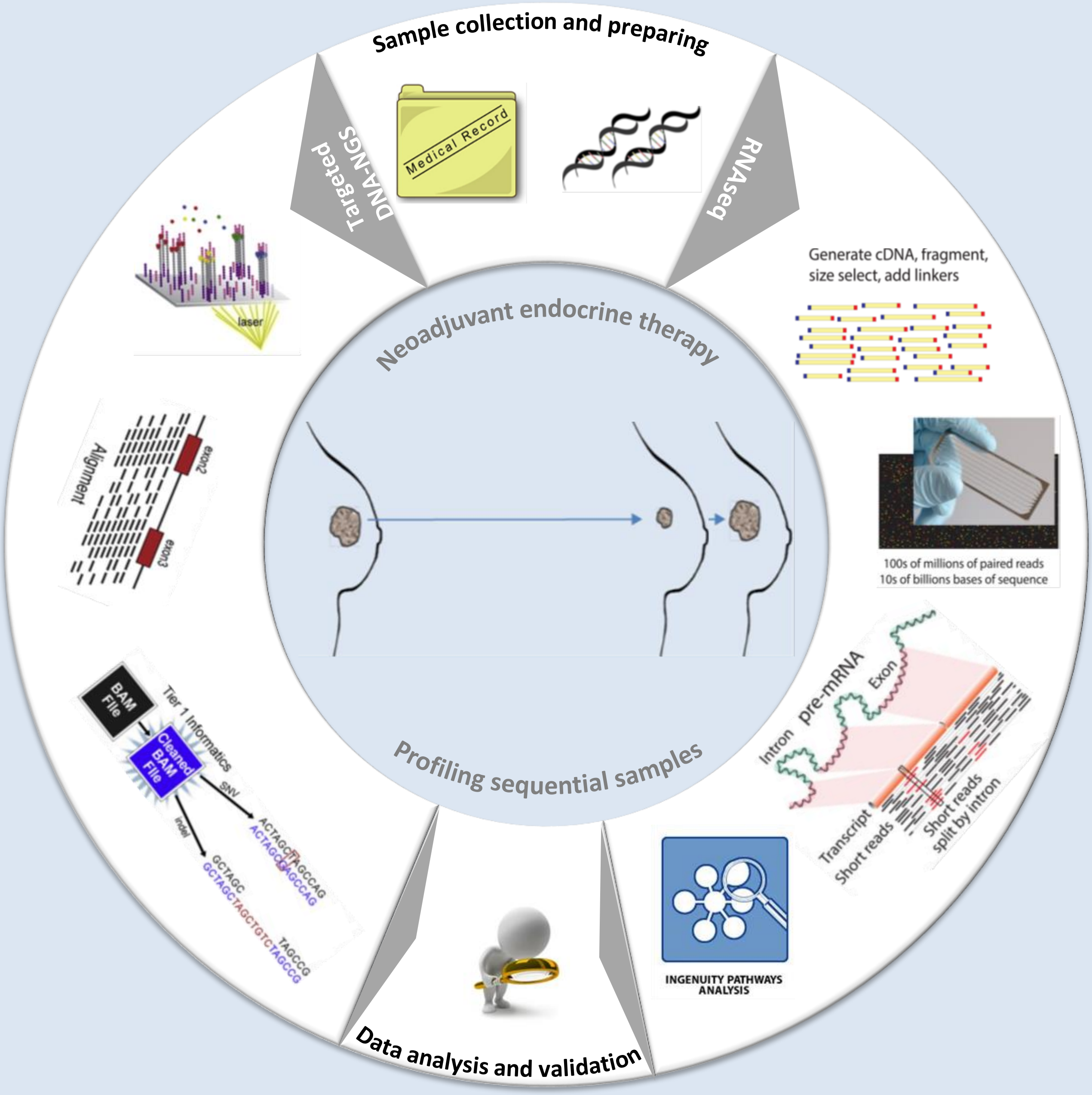

Supplement: Supplementary file 2 — Figure S1. Graphic table of contents. (PDF 561 kb) [file 13045_2018_670_MOESM2_ESM.pdf]
