# Supplementary figures and images for: Molecular profiling of hormone receptor-positive, HER2-negative breast cancers from patients treated with neoadjuvant endocrine therapy in the CARMINA 02 trial (UCBG-0609)
Source: J Hematol Oncol. 2018 Oct 11;11:124. doi: 10.1186/s13045-018-0670-9 (PMC6180434; doi:10.1186/s13045-018-0670-9)

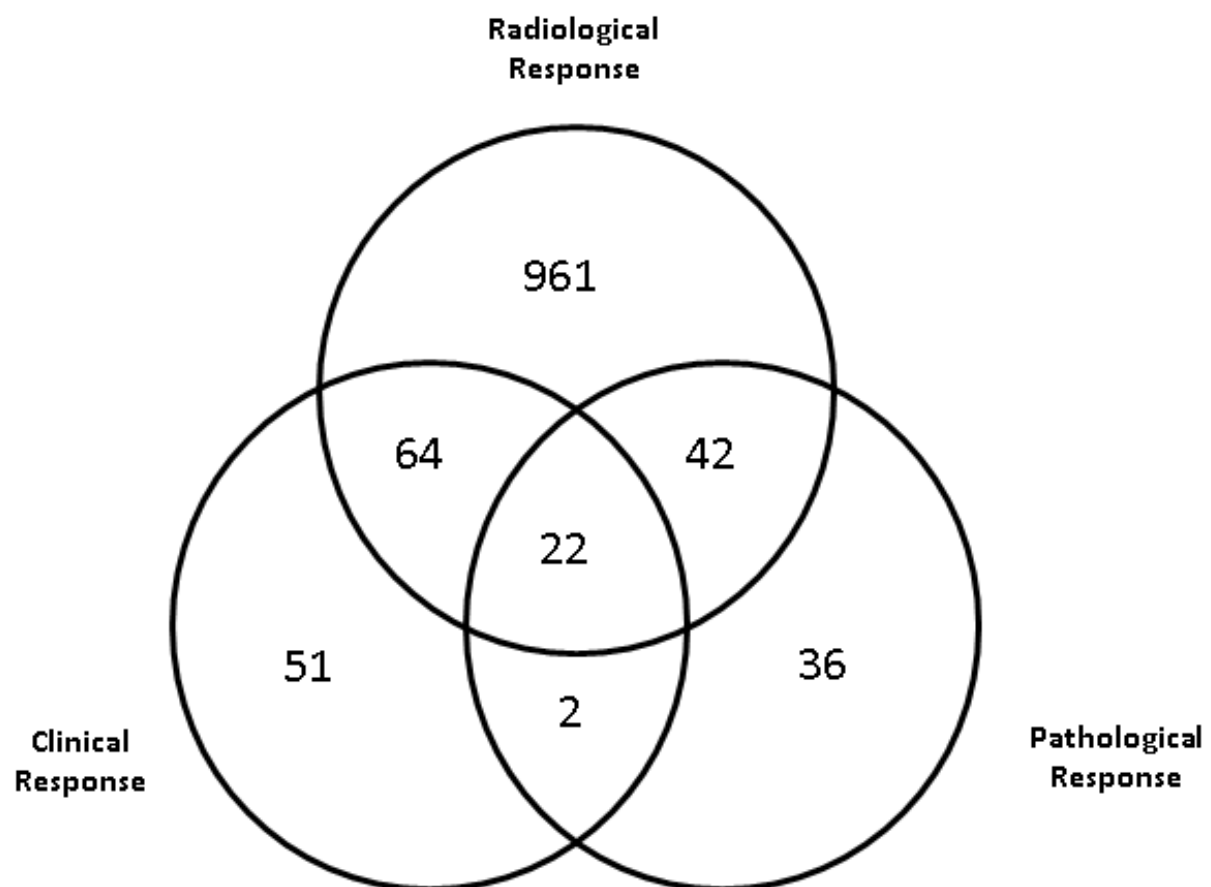

Supplement: Supplementary file 3 — Figure S2. Number of overlapping DEGs on pre- and post-NET in responders defined by three different response assessments. Venn diagram showing the total numbers and overlapping numbers of differentially expressed genes pre-NET compared to post-NET based on RNA sequencing data for responding patients using three different evaluation methods. DEGs: differentially expressed genes. (PDF 15 kb) [file 13045_2018_670_MOESM3_ESM.pdf]
