# Supplementary material for: Molecular profiling of hormone receptor-positive, HER2-negative breast cancers from patients treated with neoadjuvant endocrine therapy in the CARMINA 02 trial (UCBG-0609)
Source: J Hematol Oncol. 2018 Oct 11;11:124. doi: 10.1186/s13045-018-0670-9 (PMC6180434; doi:10.1186/s13045-018-0670-9)
Supplement: Supplementary file 4 — Table S2. Genes differentially expressed between post- and pre-NET in samples from responders and non-responders. (DOCX 82 kb) [file 13045_2018_670_MOESM4_ESM.docx]

**Table S2. Genes differentially expressed between post- and pre-NET in samples from responders and non-responders**

| **Responders** | | | **Non-responders** | | |
| --- | --- | --- | --- | --- | --- |
| **Gene ID** | **log2 FC**  **(post-NET/pre-NET)** | ***p*-value adjusted** | **Gene ID** | **log2 FC**  **(post-NET/pre-NET)** | ***p*-value adjusted** |
| **Up-regulated genes** | | | | | |
| CHGB | 2.16E+00 | 7.09E-08 | DUSP1 | 1.58E+00 | 1.04E-04 |
| VNN1 | 1.03E+00 | 1.03E-05 | KCNK3 | 1.62E+00 | 1.45E-03 |
| TOX | 1.42E+00 | 4.20E-05 | SCGN | 1.41E+00 | 7.57E-03 |
| DUSP1 | 1.38E+00 | 4.20E-05 | EGR2 | 1.28E+00 | 1.02E-02 |
| JUN | 1.02E+00 | 5.39E-05 | ZFP36 | 1.09E+00 | 1.13E-02 |
| DOC2GP | 1.59E+00 | 1.25E-04 | FOS | 1.40E+00 | 1.17E-02 |
| ZFP36 | 1.02E+00 | 1.25E-04 | NOV | 1.33E+00 | 1.17E-02 |
| POU2F2 | 1.09E+00 | 1.94E-04 | FOSB | 1.36E+00 | 1.30E-02 |
| PIK3CD | 9.39E-01 | 2.03E-04 | PTGS2 | 1.33E+00 | 1.30E-02 |
| LRRK1 | 8.31E-01 | 2.95E-04 | SULT1C2P1 | 1.33E+00 | 1.51E-02 |
| HLA-DQB2 | 1.24E+00 | 5.47E-04 | CCL3 | 1.24E+00 | 1.51E-02 |
| TRGC1 | 1.51E+00 | 5.94E-04 | NR4A1 | 1.23E+00 | 1.51E-02 |
| BCL11B | 1.38E+00 | 5.94E-04 | AF001548.5 | 1.02E+00 | 1.56E-02 |
| IPCEF1 | 1.28E+00 | 6.16E-04 | EGR1 | 1.28E+00 | 1.92E-02 |
| LCP1 | 9.97E-01 | 6.16E-04 | MKX | 1.28E+00 | 2.27E-02 |
| SMAP2 | 5.28E-01 | 6.16E-04 | MYH11 | 8.87E-01 | 2.88E-02 |
| KCNA3 | 1.38E+00 | 6.37E-04 | HAO2 | 1.17E+00 | 4.79E-02 |
| KIF21B | 1.11E+00 | 7.01E-04 | SELE | 1.09E+00 | 4.79E-02 |
| IER2 | 7.57E-01 | 8.41E-04 | CCL4 | 1.03E+00 | 5.52E-02 |
| EGR1 | 1.32E+00 | 9.04E-04 | C12orf39 | 1.16E+00 | 5.53E-02 |
| FOS | 1.41E+00 | 9.18E-04 | RIMBP2 | 1.15E+00 | 6.37E-02 |
| TMC8 | 1.17E+00 | 9.99E-04 | RP11-96C23.5 | 9.85E-01 | 6.46E-02 |
| FAM65B | 1.35E+00 | 1.04E-03 | LINC-PINT | 7.80E-01 | 7.16E-02 |
| THEMIS | 1.35E+00 | 1.07E-03 | OSM | 1.05E+00 | 7.19E-02 |
| CCL3 | 1.24E+00 | 1.07E-03 | ALB | 1.13E+00 | 7.48E-02 |
| RP11-960L18.1 | 1.47E+00 | 1.17E-03 | ST6GAL1 | 6.21E-01 | 9.30E-02 |
| CD3G | 1.36E+00 | 1.17E-03 | RNF125 | 7.13E-01 | 9.57E-02 |
| RP11-876N24.2 | 1.19E+00 | 1.17E-03 | BMP3 | 1.10E+00 | 9.93E-02 |
| TRAF3IP3 | 1.13E+00 | 1.17E-03 |  |  |  |
| NLRP1 | 8.73E-01 | 1.21E-03 |  |  |  |
| SPN | 1.13E+00 | 1.22E-03 |  |  |  |
| ST6GAL1 | 1.10E+00 | 1.24E-03 |  |  |  |
| RORB | 1.35E+00 | 1.30E-03 |  |  |  |
| CD96 | 1.23E+00 | 1.35E-03 |  |  |  |
| CD69 | 1.20E+00 | 1.35E-03 |  |  |  |
| CARD11 | 1.09E+00 | 1.35E-03 |  |  |  |
| NLRC3 | 1.03E+00 | 1.40E-03 |  |  |  |
| CD84 | 9.74E-01 | 1.40E-03 |  |  |  |
| SIRPG | 1.35E+00 | 1.41E-03 |  |  |  |
| WNT16 | 1.30E+00 | 1.41E-03 |  |  |  |
| DISC1 | 7.12E-01 | 1.41E-03 |  |  |  |
| CD5 | 1.20E+00 | 1.43E-03 |  |  |  |
| RASSF5 | 9.53E-01 | 1.43E-03 |  |  |  |
| ZNF831 | 1.36E+00 | 1.47E-03 |  |  |  |
| KLRD1 | 9.68E-01 | 1.56E-03 |  |  |  |
| RP11-94L15.2 | 1.22E+00 | 1.62E-03 |  |  |  |
| TAGAP | 1.01E+00 | 1.62E-03 |  |  |  |
| RASAL3 | 1.03E+00 | 1.68E-03 |  |  |  |
| CD40LG | 1.29E+00 | 1.75E-03 |  |  |  |
| CIITA | 6.25E-01 | 1.83E-03 |  |  |  |
| TRAV1-1 | 1.42E+00 | 1.84E-03 |  |  |  |
| PARP15 | 1.29E+00 | 1.86E-03 |  |  |  |
| BANK1 | 1.39E+00 | 1.88E-03 |  |  |  |
| CD8B | 1.18E+00 | 1.88E-03 |  |  |  |
| CD8A | 1.17E+00 | 1.96E-03 |  |  |  |
| CAMK4 | 1.14E+00 | 2.00E-03 |  |  |  |
| SH2D1A | 1.28E+00 | 2.00E-03 |  |  |  |
| IKZF1 | 1.10E+00 | 2.05E-03 |  |  |  |
| EOMES | 1.29E+00 | 2.14E-03 |  |  |  |
| PYHIN1 | 1.28E+00 | 2.25E-03 |  |  |  |
| NUGGC | 1.19E+00 | 2.25E-03 |  |  |  |
| KLRC4-KLRK1 | 1.18E+00 | 2.30E-03 |  |  |  |
| CYR61 | 1.10E+00 | 2.37E-03 |  |  |  |
| RCSD1 | 9.13E-01 | 2.42E-03 |  |  |  |
| CCDC88B | 9.38E-01 | 2.44E-03 |  |  |  |
| KLRK1 | 1.18E+00 | 2.50E-03 |  |  |  |
| FASLG | 1.17E+00 | 2.50E-03 |  |  |  |
| GRAP2 | 1.14E+00 | 2.61E-03 |  |  |  |
| LAX1 | 1.26E+00 | 2.65E-03 |  |  |  |
| ITK | 1.23E+00 | 2.73E-03 |  |  |  |
| MAP4K1 | 1.04E+00 | 2.79E-03 |  |  |  |
| IL12RB2 | 1.19E+00 | 2.81E-03 |  |  |  |
| INPP5D | 8.93E-01 | 2.88E-03 |  |  |  |
| AL122127.25 | 1.36E+00 | 2.90E-03 |  |  |  |
| DHRS9 | 1.10E+00 | 2.97E-03 |  |  |  |
| IL21R | 1.06E+00 | 2.97E-03 |  |  |  |
| NCAM1 | 1.22E+00 | 3.06E-03 |  |  |  |
| HLA-DOA | 8.69E-01 | 3.10E-03 |  |  |  |
| TRAV8-3 | 1.30E+00 | 3.20E-03 |  |  |  |
| PRKCB | 1.21E+00 | 3.21E-03 |  |  |  |
| JUNB | 8.10E-01 | 3.27E-03 |  |  |  |
| TIGIT | 1.24E+00 | 3.27E-03 |  |  |  |
| CCDC17 | 7.14E-01 | 3.27E-03 |  |  |  |
| P2RY10 | 1.26E+00 | 3.34E-03 |  |  |  |
| GFI1 | 1.12E+00 | 3.34E-03 |  |  |  |
| PLAG1 | 9.68E-01 | 3.34E-03 |  |  |  |
| HIVEP2 | 6.86E-01 | 3.34E-03 |  |  |  |
| IGLL1 | 1.20E+00 | 3.38E-03 |  |  |  |
| HLA-DOB | 1.15E+00 | 3.38E-03 |  |  |  |
| SAMD3 | 1.05E+00 | 3.51E-03 |  |  |  |
| LCK | 1.18E+00 | 3.58E-03 |  |  |  |
| ZAP70 | 1.14E+00 | 3.60E-03 |  |  |  |
| CD3E | 1.19E+00 | 3.76E-03 |  |  |  |
| IL22RA2 | 1.33E+00 | 3.95E-03 |  |  |  |
| CD7 | 1.12E+00 | 4.19E-03 |  |  |  |
| PIM2 | 8.45E-01 | 4.26E-03 |  |  |  |
| ANKRD44 | 7.88E-01 | 4.26E-03 |  |  |  |
| FUT6 | 1.17E+00 | 4.51E-03 |  |  |  |
| CD247 | 1.11E+00 | 4.57E-03 |  |  |  |
| KIAA0125 | 1.31E+00 | 4.63E-03 |  |  |  |
| WDFY4 | 1.01E+00 | 4.66E-03 |  |  |  |
| NFATC2 | 7.65E-01 | 4.66E-03 |  |  |  |
| IL21R-AS1 | 1.02E+00 | 4.69E-03 |  |  |  |
| LAT | 7.55E-01 | 4.69E-03 |  |  |  |
| DTHD1 | 1.26E+00 | 4.73E-03 |  |  |  |
| TLR10 | 1.20E+00 | 4.73E-03 |  |  |  |
| RP11-215G15.5 | 7.88E-01 | 4.75E-03 |  |  |  |
| EDIL3 | 1.16E+00 | 4.82E-03 |  |  |  |
| TDRD12 | 1.25E+00 | 5.10E-03 |  |  |  |
| SLAMF1 | 1.15E+00 | 5.10E-03 |  |  |  |
| GPR55 | 1.14E+00 | 5.10E-03 |  |  |  |
| RP11-455F5.5 | 9.95E-01 | 5.10E-03 |  |  |  |
| RP11-689B22.2 | 9.19E-01 | 5.10E-03 |  |  |  |
| ZCCHC18 | 8.31E-01 | 5.10E-03 |  |  |  |
| SLAIN1 | 1.20E+00 | 5.35E-03 |  |  |  |
| ACAP1 | 1.10E+00 | 5.36E-03 |  |  |  |
| ADRB2 | 8.79E-01 | 5.37E-03 |  |  |  |
| TFEB | 5.47E-01 | 5.37E-03 |  |  |  |
| ZNF80 | 1.12E+00 | 5.44E-03 |  |  |  |
| PLCG2 | 8.74E-01 | 5.45E-03 |  |  |  |
| MIAT | 1.01E+00 | 5.77E-03 |  |  |  |
| LINC00426 | 1.13E+00 | 5.87E-03 |  |  |  |
| APOBEC3D | 7.16E-01 | 5.87E-03 |  |  |  |
| ITGB2-AS1 | 1.02E+00 | 5.87E-03 |  |  |  |
| FAM129C | 1.26E+00 | 5.88E-03 |  |  |  |
| CXCR6 | 1.01E+00 | 5.93E-03 |  |  |  |
| APBA2 | 9.77E-01 | 5.93E-03 |  |  |  |
| FAM107B | 8.62E-01 | 5.93E-03 |  |  |  |
| TNFAIP3 | 8.35E-01 | 5.93E-03 |  |  |  |
| SPOCK2 | 1.06E+00 | 6.04E-03 |  |  |  |
| FAM19A1 | 1.17E+00 | 6.09E-03 |  |  |  |
| SNAP91 | 1.26E+00 | 6.26E-03 |  |  |  |
| CTSW | 1.05E+00 | 6.34E-03 |  |  |  |
| LINC00936 | 7.04E-01 | 6.34E-03 |  |  |  |
| C11orf21 | 1.02E+00 | 6.46E-03 |  |  |  |
| SH3KBP1 | 6.11E-01 | 6.53E-03 |  |  |  |
| SLAMF6 | 1.16E+00 | 6.55E-03 |  |  |  |
| C6orf58 | 1.21E+00 | 6.74E-03 |  |  |  |
| CD6 | 1.07E+00 | 6.74E-03 |  |  |  |
| LINC00612 | 8.52E-01 | 6.90E-03 |  |  |  |
| HMHA1 | 7.16E-01 | 6.91E-03 |  |  |  |
| KLRC4 | 1.15E+00 | 7.16E-03 |  |  |  |
| PRKCQ | 1.12E+00 | 7.20E-03 |  |  |  |
| PCED1B-AS1 | 9.09E-01 | 7.26E-03 |  |  |  |
| PLEK | 9.17E-01 | 7.28E-03 |  |  |  |
| NAPSB | 1.00E+00 | 7.29E-03 |  |  |  |
| CTD-2020K17.3 | 7.71E-01 | 7.32E-03 |  |  |  |
| CORO1A | 9.20E-01 | 7.37E-03 |  |  |  |
| SEL1L3 | 1.01E+00 | 7.52E-03 |  |  |  |
| FAM117A | 6.38E-01 | 7.69E-03 |  |  |  |
| PTPRCAP | 9.94E-01 | 8.08E-03 |  |  |  |
| SCML4 | 1.13E+00 | 8.12E-03 |  |  |  |
| CTLA4 | 1.09E+00 | 8.12E-03 |  |  |  |
| C16orf54 | 1.05E+00 | 8.12E-03 |  |  |  |
| TRAT1 | 1.16E+00 | 8.13E-03 |  |  |  |
| TBC1D10C | 9.90E-01 | 8.23E-03 |  |  |  |
| PPARA | 8.46E-01 | 8.33E-03 |  |  |  |
| PPP1R16B | 1.00E+00 | 8.34E-03 |  |  |  |
| BTLA | 1.20E+00 | 8.35E-03 |  |  |  |
| CD2 | 1.06E+00 | 8.41E-03 |  |  |  |
| AKNA | 8.10E-01 | 8.51E-03 |  |  |  |
| KRT2 | 1.23E+00 | 8.56E-03 |  |  |  |
| TNFRSF9 | 1.15E+00 | 8.56E-03 |  |  |  |
| P2RX5 | 1.12E+00 | 8.56E-03 |  |  |  |
| TNF | 8.95E-01 | 8.56E-03 |  |  |  |
| KLHL6 | 9.74E-01 | 8.56E-03 |  |  |  |
| PRDM8 | 9.04E-01 | 8.56E-03 |  |  |  |
| LINC00926 | 1.01E+00 | 8.66E-03 |  |  |  |
| ABCB4 | 7.57E-01 | 8.66E-03 |  |  |  |
| SH2D3C | 6.25E-01 | 8.66E-03 |  |  |  |
| TSPAN32 | 9.85E-01 | 8.70E-03 |  |  |  |
| CTA-211A9.5 | 9.41E-01 | 8.70E-03 |  |  |  |
| SYNE1 | 5.96E-01 | 8.70E-03 |  |  |  |
| CD1E | 1.13E+00 | 8.90E-03 |  |  |  |
| ARHGAP9 | 8.04E-01 | 8.95E-03 |  |  |  |
| CXCR3 | 1.02E+00 | 9.06E-03 |  |  |  |
| IL16 | 8.01E-01 | 9.09E-03 |  |  |  |
| CD244 | 1.01E+00 | 9.25E-03 |  |  |  |
| LY9 | 1.14E+00 | 9.29E-03 |  |  |  |
| FOXF1 | 9.75E-01 | 9.38E-03 |  |  |  |
| TRAC | 9.28E-01 | 9.38E-03 |  |  |  |
| NKG7 | 1.06E+00 | 9.46E-03 |  |  |  |
| STAT4 | 9.80E-01 | 9.56E-03 |  |  |  |
| STK17B | 7.57E-01 | 9.73E-03 |  |  |  |
| IRF4 | 1.14E+00 | 9.78E-03 |  |  |  |
| PATL2 | 9.41E-01 | 9.99E-03 |  |  |  |
| POU2AF1 | 1.17E+00 | 1.02E-02 |  |  |  |
| CCDC136 | 8.42E-01 | 1.02E-02 |  |  |  |
| TTC24 | 1.20E+00 | 1.03E-02 |  |  |  |
| FCRL3 | 1.20E+00 | 1.03E-02 |  |  |  |
| RP11-356I2.4 | 9.36E-01 | 1.03E-02 |  |  |  |
| ICOS | 1.07E+00 | 1.03E-02 |  |  |  |
| SELL | 1.09E+00 | 1.05E-02 |  |  |  |
| POU3F1 | 1.03E+00 | 1.06E-02 |  |  |  |
| APOBEC3G | 6.77E-01 | 1.06E-02 |  |  |  |
| ITGAD | 1.05E+00 | 1.07E-02 |  |  |  |
| ZNF683 | 1.07E+00 | 1.10E-02 |  |  |  |
| XCL1 | 9.96E-01 | 1.10E-02 |  |  |  |
| GPR155 | 6.43E-01 | 1.10E-02 |  |  |  |
| PIK3IP1 | 5.55E-01 | 1.10E-02 |  |  |  |
| LPAL2 | 5.81E-01 | 1.12E-02 |  |  |  |
| FMNL1 | 6.94E-01 | 1.12E-02 |  |  |  |
| LINC00092 | 9.93E-01 | 1.15E-02 |  |  |  |
| IL2RG | 1.02E+00 | 1.15E-02 |  |  |  |
| IFNG-AS1 | 1.16E+00 | 1.18E-02 |  |  |  |
| NEFL | 1.16E+00 | 1.19E-02 |  |  |  |
| JAK3 | 9.68E-01 | 1.21E-02 |  |  |  |
| FOXP3 | 7.97E-01 | 1.21E-02 |  |  |  |
| SIT1 | 1.10E+00 | 1.22E-02 |  |  |  |
| SNX20 | 9.57E-01 | 1.22E-02 |  |  |  |
| RASGRP2 | 1.02E+00 | 1.23E-02 |  |  |  |
| IL12RB1 | 8.94E-01 | 1.23E-02 |  |  |  |
| CUBN | 7.65E-01 | 1.23E-02 |  |  |  |
| POU6F1 | 5.77E-01 | 1.23E-02 |  |  |  |
| LRMP | 9.31E-01 | 1.25E-02 |  |  |  |
| TMEM156 | 1.06E+00 | 1.25E-02 |  |  |  |
| CCR2 | 1.01E+00 | 1.25E-02 |  |  |  |
| CDHR1 | 1.11E+00 | 1.25E-02 |  |  |  |
| FCRL6 | 1.00E+00 | 1.25E-02 |  |  |  |
| TRBV4-1 | 1.16E+00 | 1.26E-02 |  |  |  |
| ASB2 | 9.63E-01 | 1.26E-02 |  |  |  |
| C20orf203 | 9.33E-01 | 1.26E-02 |  |  |  |
| DOCK2 | 8.52E-01 | 1.30E-02 |  |  |  |
| MS4A1 | 1.18E+00 | 1.31E-02 |  |  |  |
| PLEKHG7 | 1.15E+00 | 1.31E-02 |  |  |  |
| SPIB | 1.14E+00 | 1.31E-02 |  |  |  |
| GAB3 | 7.11E-01 | 1.34E-02 |  |  |  |
| FYN | 6.89E-01 | 1.34E-02 |  |  |  |
| LTA | 1.04E+00 | 1.36E-02 |  |  |  |
| CLEC2D | 5.63E-01 | 1.39E-02 |  |  |  |
| RNF166 | 5.53E-01 | 1.39E-02 |  |  |  |
| PRDM1 | 6.47E-01 | 1.42E-02 |  |  |  |
| TLR7 | 7.00E-01 | 1.43E-02 |  |  |  |
| DENND1C | 5.91E-01 | 1.43E-02 |  |  |  |
| ETS1 | 6.87E-01 | 1.44E-02 |  |  |  |
| UBASH3A | 1.08E+00 | 1.45E-02 |  |  |  |
| MYO7B | 9.36E-01 | 1.45E-02 |  |  |  |
| PLCL2 | 7.66E-01 | 1.45E-02 |  |  |  |
| ELAC1 | 5.27E-01 | 1.46E-02 |  |  |  |
| TNR | 1.16E+00 | 1.47E-02 |  |  |  |
| TRAV2 | 1.16E+00 | 1.47E-02 |  |  |  |
| L3MBTL3 | 6.46E-01 | 1.47E-02 |  |  |  |
| C12orf79 | 8.21E-01 | 1.48E-02 |  |  |  |
| FAM13A-AS1 | 6.91E-01 | 1.48E-02 |  |  |  |
| CD207 | 1.14E+00 | 1.49E-02 |  |  |  |
| CD79A | 1.14E+00 | 1.49E-02 |  |  |  |
| RP11-571M6.8 | 7.51E-01 | 1.50E-02 |  |  |  |
| P2RY13 | 8.61E-01 | 1.53E-02 |  |  |  |
| NDP | 1.15E+00 | 1.57E-02 |  |  |  |
| PTK2B | 5.62E-01 | 1.58E-02 |  |  |  |
| KLRC1 | 1.09E+00 | 1.59E-02 |  |  |  |
| STAP1 | 1.06E+00 | 1.59E-02 |  |  |  |
| WAS | 7.78E-01 | 1.59E-02 |  |  |  |
| RNF125 | 7.00E-01 | 1.59E-02 |  |  |  |
| CITED2 | 6.10E-01 | 1.59E-02 |  |  |  |
| NDST3 | 1.07E+00 | 1.60E-02 |  |  |  |
| NPL | 6.45E-01 | 1.60E-02 |  |  |  |
| ALOX5 | 8.34E-01 | 1.60E-02 |  |  |  |
| MIR766 | 5.81E-01 | 1.61E-02 |  |  |  |
| CTC-378H22.1 | 9.60E-01 | 1.63E-02 |  |  |  |
| CD4 | 7.37E-01 | 1.63E-02 |  |  |  |
| PLCB2 | 7.21E-01 | 1.63E-02 |  |  |  |
| PYGM | 9.19E-01 | 1.65E-02 |  |  |  |
| L1CAM | 6.81E-01 | 1.65E-02 |  |  |  |
| AFF2 | 9.53E-01 | 1.66E-02 |  |  |  |
| ITGAL | 8.92E-01 | 1.67E-02 |  |  |  |
| HLA-DPB1 | 6.82E-01 | 1.69E-02 |  |  |  |
| SELE | 9.59E-01 | 1.73E-02 |  |  |  |
| BIN1 | 7.75E-01 | 1.75E-02 |  |  |  |
| TRAV13-2 | 1.13E+00 | 1.75E-02 |  |  |  |
| HTR7 | 9.56E-01 | 1.75E-02 |  |  |  |
| TBX21 | 9.87E-01 | 1.76E-02 |  |  |  |
| PIGR | 1.09E+00 | 1.77E-02 |  |  |  |
| PDCD1 | 1.05E+00 | 1.77E-02 |  |  |  |
| CXCL3 | 9.85E-01 | 1.77E-02 |  |  |  |
| ERMN | 9.22E-01 | 1.77E-02 |  |  |  |
| TCF7 | 9.00E-01 | 1.77E-02 |  |  |  |
| ZC3H12D | 8.88E-01 | 1.77E-02 |  |  |  |
| ARRDC5 | 8.73E-01 | 1.77E-02 |  |  |  |
| PAG1 | 7.66E-01 | 1.77E-02 |  |  |  |
| BTN3A1 | 5.62E-01 | 1.77E-02 |  |  |  |
| ADCY7 | 6.36E-01 | 1.79E-02 |  |  |  |
| CCL5 | 8.90E-01 | 1.79E-02 |  |  |  |
| MPP1 | 5.11E-01 | 1.79E-02 |  |  |  |
| GZMM | 9.45E-01 | 1.80E-02 |  |  |  |
| DBP | 6.55E-01 | 1.80E-02 |  |  |  |
| RP11-148O21.3 | 1.13E+00 | 1.80E-02 |  |  |  |
| GCGR | 1.10E+00 | 1.80E-02 |  |  |  |
| LINC00996 | 1.00E+00 | 1.80E-02 |  |  |  |
| TRAV13-1 | 1.05E+00 | 1.81E-02 |  |  |  |
| IRAK2 | 6.47E-01 | 1.81E-02 |  |  |  |
| ARHGAP25 | 8.25E-01 | 1.83E-02 |  |  |  |
| CXCR4 | 7.58E-01 | 1.83E-02 |  |  |  |
| CD1C | 1.07E+00 | 1.84E-02 |  |  |  |
| PNOC | 1.07E+00 | 1.84E-02 |  |  |  |
| TESPA1 | 9.74E-01 | 1.84E-02 |  |  |  |
| LPAR5 | 6.44E-01 | 1.84E-02 |  |  |  |
| PNMA3 | 1.00E+00 | 1.84E-02 |  |  |  |
| ARHGAP26 | 7.46E-01 | 1.84E-02 |  |  |  |
| MAPK4 | 1.09E+00 | 1.84E-02 |  |  |  |
| CAMK1D | 6.64E-01 | 1.88E-02 |  |  |  |
| TIFAB | 1.06E+00 | 1.90E-02 |  |  |  |
| BACH2 | 9.15E-01 | 1.90E-02 |  |  |  |
| BIRC3 | 9.32E-01 | 1.91E-02 |  |  |  |
| SIAH3 | 1.12E+00 | 1.94E-02 |  |  |  |
| ZFR2 | 1.10E+00 | 1.94E-02 |  |  |  |
| SLC12A3 | 1.03E+00 | 1.94E-02 |  |  |  |
| NCR3 | 1.02E+00 | 1.97E-02 |  |  |  |
| C5orf20 | 1.05E+00 | 1.98E-02 |  |  |  |
| ANKRD34A | 7.26E-01 | 1.98E-02 |  |  |  |
| HLA-DPA1 | 6.37E-01 | 1.98E-02 |  |  |  |
| PTPRC | 9.35E-01 | 2.02E-02 |  |  |  |
| CD3D | 1.01E+00 | 2.02E-02 |  |  |  |
| LINC00861 | 1.00E+00 | 2.04E-02 |  |  |  |
| IGLV1-51 | 1.01E+00 | 2.05E-02 |  |  |  |
| FAM78A | 7.56E-01 | 2.05E-02 |  |  |  |
| CD79B | 1.05E+00 | 2.08E-02 |  |  |  |
| IFNG | 1.09E+00 | 2.08E-02 |  |  |  |
| RP11-544L8__B.4 | 1.03E+00 | 2.09E-02 |  |  |  |
| EVI2B | 8.37E-01 | 2.09E-02 |  |  |  |
| MEI1 | 8.25E-01 | 2.09E-02 |  |  |  |
| BLK | 1.10E+00 | 2.10E-02 |  |  |  |
| CCR7 | 1.00E+00 | 2.10E-02 |  |  |  |
| GPR174 | 1.05E+00 | 2.13E-02 |  |  |  |
| CLNK | 1.08E+00 | 2.15E-02 |  |  |  |
| APBB1IP | 7.55E-01 | 2.16E-02 |  |  |  |
| LINC00578 | 1.04E+00 | 2.16E-02 |  |  |  |
| XCL2 | 9.95E-01 | 2.16E-02 |  |  |  |
| AC006129.2 | 1.07E+00 | 2.17E-02 |  |  |  |
| CCR4 | 1.01E+00 | 2.18E-02 |  |  |  |
| CD97 | 7.28E-01 | 2.20E-02 |  |  |  |
| WNT10A | 9.88E-01 | 2.24E-02 |  |  |  |
| TNIP3 | 1.05E+00 | 2.24E-02 |  |  |  |
| VCAM1 | 8.77E-01 | 2.28E-02 |  |  |  |
| FCER2 | 1.10E+00 | 2.28E-02 |  |  |  |
| PVRIG | 7.22E-01 | 2.31E-02 |  |  |  |
| GZMK | 1.00E+00 | 2.34E-02 |  |  |  |
| SIGLEC8 | 7.42E-01 | 2.34E-02 |  |  |  |
| CXorf21 | 7.34E-01 | 2.36E-02 |  |  |  |
| GCM1 | 1.08E+00 | 2.36E-02 |  |  |  |
| PDZRN4 | 9.97E-01 | 2.36E-02 |  |  |  |
| GZMA | 9.29E-01 | 2.41E-02 |  |  |  |
| MROH7 | 7.57E-01 | 2.42E-02 |  |  |  |
| RLTPR | 9.50E-01 | 2.45E-02 |  |  |  |
| LRRK2 | 9.19E-01 | 2.51E-02 |  |  |  |
| TRDC | 8.33E-01 | 2.59E-02 |  |  |  |
| CCR6 | 8.54E-01 | 2.60E-02 |  |  |  |
| IL32 | 6.92E-01 | 2.60E-02 |  |  |  |
| SLCO5A1 | 1.04E+00 | 2.60E-02 |  |  |  |
| CD19 | 1.08E+00 | 2.63E-02 |  |  |  |
| LTB | 1.02E+00 | 2.64E-02 |  |  |  |
| CST7 | 8.88E-01 | 2.65E-02 |  |  |  |
| KIAA1683 | 6.37E-01 | 2.65E-02 |  |  |  |
| ACY3 | 1.06E+00 | 2.68E-02 |  |  |  |
| CXCL13 | 1.05E+00 | 2.68E-02 |  |  |  |
| RP11-166B2.1 | 1.03E+00 | 2.68E-02 |  |  |  |
| RP3-395M20.9 | 9.81E-01 | 2.68E-02 |  |  |  |
| CXorf65 | 9.62E-01 | 2.68E-02 |  |  |  |
| LCP2 | 6.90E-01 | 2.68E-02 |  |  |  |
| SLC6A12 | 8.92E-01 | 2.68E-02 |  |  |  |
| TEC | 6.05E-01 | 2.71E-02 |  |  |  |
| AOAH | 8.20E-01 | 2.73E-02 |  |  |  |
| PTPN7 | 8.82E-01 | 2.74E-02 |  |  |  |
| ZBED2 | 1.05E+00 | 2.79E-02 |  |  |  |
| NCF1 | 7.98E-01 | 2.79E-02 |  |  |  |
| PRKCQ-AS1 | 1.01E+00 | 2.81E-02 |  |  |  |
| CTD-2269F5.1 | 9.68E-01 | 2.81E-02 |  |  |  |
| KCND1 | 5.76E-01 | 2.83E-02 |  |  |  |
| TRBV4-2 | 1.05E+00 | 2.84E-02 |  |  |  |
| LINGO3 | 9.53E-01 | 2.88E-02 |  |  |  |
| PLEKHF1 | 7.13E-01 | 2.90E-02 |  |  |  |
| IL12B | 1.02E+00 | 2.90E-02 |  |  |  |
| HMCN2 | 9.98E-01 | 2.90E-02 |  |  |  |
| DOCK8 | 7.08E-01 | 2.90E-02 |  |  |  |
| LINC00511 | 8.24E-01 | 2.90E-02 |  |  |  |
| THADA | 5.57E-01 | 2.90E-02 |  |  |  |
| TNFRSF17 | 1.03E+00 | 2.91E-02 |  |  |  |
| GPR114 | 9.57E-01 | 2.91E-02 |  |  |  |
| ZFP36L2 | 6.44E-01 | 2.91E-02 |  |  |  |
| ARHGAP30 | 7.13E-01 | 2.91E-02 |  |  |  |
| IKZF3 | 1.01E+00 | 2.94E-02 |  |  |  |
| XCR1 | 1.05E+00 | 2.94E-02 |  |  |  |
| F3 | 8.48E-01 | 2.97E-02 |  |  |  |
| TRAF1 | 6.23E-01 | 2.97E-02 |  |  |  |
| GBP5 | 9.33E-01 | 3.00E-02 |  |  |  |
| PRF1 | 8.95E-01 | 3.00E-02 |  |  |  |
| NRN1L | 6.33E-01 | 3.00E-02 |  |  |  |
| PLD4 | 8.20E-01 | 3.02E-02 |  |  |  |
| RGAG4 | 7.79E-01 | 3.02E-02 |  |  |  |
| NBPF13P | 6.31E-01 | 3.03E-02 |  |  |  |
| FCRL5 | 1.05E+00 | 3.03E-02 |  |  |  |
| TRAV36DV7 | 1.04E+00 | 3.07E-02 |  |  |  |
| CCDC146 | 5.08E-01 | 3.08E-02 |  |  |  |
| SPNS3 | 8.11E-01 | 3.09E-02 |  |  |  |
| NHSL2 | 7.19E-01 | 3.09E-02 |  |  |  |
| LY75 | 6.94E-01 | 3.09E-02 |  |  |  |
| BLNK | 5.53E-01 | 3.10E-02 |  |  |  |
| FTCD | 8.36E-01 | 3.11E-02 |  |  |  |
| LINC00892 | 9.49E-01 | 3.14E-02 |  |  |  |
| RP1-249H1.4 | 6.28E-01 | 3.14E-02 |  |  |  |
| PIK3CG | 8.59E-01 | 3.15E-02 |  |  |  |
| MYH3 | 5.59E-01 | 3.16E-02 |  |  |  |
| GZMH | 9.29E-01 | 3.18E-02 |  |  |  |
| SPINK2 | 1.02E+00 | 3.19E-02 |  |  |  |
| CXXC11 | 1.05E+00 | 3.21E-02 |  |  |  |
| SERPINB9 | 6.25E-01 | 3.21E-02 |  |  |  |
| CILP2 | 9.21E-01 | 3.26E-02 |  |  |  |
| TRAV3 | 1.05E+00 | 3.26E-02 |  |  |  |
| TRAV4 | 1.02E+00 | 3.26E-02 |  |  |  |
| TNRC6C-AS1 | 6.01E-01 | 3.28E-02 |  |  |  |
| PTGS2 | 9.80E-01 | 3.31E-02 |  |  |  |
| TOX2 | 8.75E-01 | 3.31E-02 |  |  |  |
| FYB | 7.98E-01 | 3.31E-02 |  |  |  |
| DGKA | 6.22E-01 | 3.33E-02 |  |  |  |
| TRAV6 | 1.03E+00 | 3.36E-02 |  |  |  |
| GLIS3 | 8.24E-01 | 3.37E-02 |  |  |  |
| FGD2 | 7.50E-01 | 3.37E-02 |  |  |  |
| BCL11A | 1.00E+00 | 3.39E-02 |  |  |  |
| VNN2 | 8.39E-01 | 3.41E-02 |  |  |  |
| C20orf197 | 7.52E-01 | 3.41E-02 |  |  |  |
| FOSB | 1.03E+00 | 3.44E-02 |  |  |  |
| FER1L5 | 1.02E+00 | 3.44E-02 |  |  |  |
| RASAL1 | 1.00E+00 | 3.45E-02 |  |  |  |
| RP11-511B23.2 | 9.69E-01 | 3.45E-02 |  |  |  |
| EPHB6 | 8.34E-01 | 3.45E-02 |  |  |  |
| CTD-2020K17.1 | 7.61E-01 | 3.45E-02 |  |  |  |
| NCF1C | 7.55E-01 | 3.45E-02 |  |  |  |
| PIK3R6 | 5.69E-01 | 3.45E-02 |  |  |  |
| TNFRSF13B | 1.03E+00 | 3.46E-02 |  |  |  |
| CD52 | 8.53E-01 | 3.46E-02 |  |  |  |
| GBA3 | 1.03E+00 | 3.47E-02 |  |  |  |
| IDO2 | 1.02E+00 | 3.48E-02 |  |  |  |
| LILRA4 | 9.19E-01 | 3.50E-02 |  |  |  |
| UGT2B4 | 1.03E+00 | 3.52E-02 |  |  |  |
| FCRLA | 1.03E+00 | 3.56E-02 |  |  |  |
| ARHGAP15 | 7.60E-01 | 3.56E-02 |  |  |  |
| TTYH2 | 6.14E-01 | 3.56E-02 |  |  |  |
| PSTPIP1 | 8.20E-01 | 3.65E-02 |  |  |  |
| INSL3 | 9.73E-01 | 3.67E-02 |  |  |  |
| FLT3LG | 5.58E-01 | 3.69E-02 |  |  |  |
| FAM13A | 6.25E-01 | 3.71E-02 |  |  |  |
| CD74 | 6.17E-01 | 3.74E-02 |  |  |  |
| ITM2C | 7.23E-01 | 3.74E-02 |  |  |  |
| IGLV4-60 | 1.02E+00 | 3.80E-02 |  |  |  |
| TRAV26-1 | 1.02E+00 | 3.82E-02 |  |  |  |
| TRAV29DV5 | 1.01E+00 | 3.82E-02 |  |  |  |
| LIPC | 8.81E-01 | 3.82E-02 |  |  |  |
| PTCH2 | 6.51E-01 | 3.85E-02 |  |  |  |
| ABCB1 | 7.52E-01 | 3.85E-02 |  |  |  |
| LYL1 | 6.74E-01 | 3.86E-02 |  |  |  |
| WIPF1 | 6.32E-01 | 3.87E-02 |  |  |  |
| RGS1 | 8.67E-01 | 3.88E-02 |  |  |  |
| RP11-834C11.7 | 6.92E-01 | 3.88E-02 |  |  |  |
| VENTX | 7.14E-01 | 3.89E-02 |  |  |  |
| CD38 | 9.66E-01 | 3.92E-02 |  |  |  |
| ABCC4 | 7.69E-01 | 3.94E-02 |  |  |  |
| CTD-2020K17.4 | 5.87E-01 | 3.97E-02 |  |  |  |
| COL6A4P2 | 8.43E-01 | 3.99E-02 |  |  |  |
| ITIH1 | 9.90E-01 | 4.03E-02 |  |  |  |
| APOBEC3C | 6.32E-01 | 4.06E-02 |  |  |  |
| ANXA2R | 6.49E-01 | 4.08E-02 |  |  |  |
| TRAV38-2DV8 | 1.01E+00 | 4.10E-02 |  |  |  |
| LAMP3 | 8.97E-01 | 4.12E-02 |  |  |  |
| TRAV25 | 1.01E+00 | 4.13E-02 |  |  |  |
| HLA-DMA | 6.18E-01 | 4.13E-02 |  |  |  |
| RP11-118B22.4 | 8.55E-01 | 4.17E-02 |  |  |  |
| SLC30A8 | 1.01E+00 | 4.19E-02 |  |  |  |
| CCR5 | 7.89E-01 | 4.24E-02 |  |  |  |
| TMIGD2 | 9.73E-01 | 4.26E-02 |  |  |  |
| OCM | 6.73E-01 | 4.26E-02 |  |  |  |
| FCRL4 | 1.01E+00 | 4.30E-02 |  |  |  |
| TRAV23DV6 | 9.92E-01 | 4.30E-02 |  |  |  |
| TRAV35 | 9.82E-01 | 4.30E-02 |  |  |  |
| KCNG1 | 1.00E+00 | 4.30E-02 |  |  |  |
| RP11-114M1.1 | 9.93E-01 | 4.30E-02 |  |  |  |
| NOG | 9.52E-01 | 4.30E-02 |  |  |  |
| FGR | 6.11E-01 | 4.30E-02 |  |  |  |
| SEMA4D | 5.29E-01 | 4.30E-02 |  |  |  |
| LINC00944 | 9.29E-01 | 4.32E-02 |  |  |  |
| FLI1 | 6.22E-01 | 4.32E-02 |  |  |  |
| LGALS2 | 9.32E-01 | 4.34E-02 |  |  |  |
| TRBC2 | 8.75E-01 | 4.34E-02 |  |  |  |
| RP11-796G6.2 | 9.53E-01 | 4.35E-02 |  |  |  |
| IGFLR1 | 5.97E-01 | 4.43E-02 |  |  |  |
| LIMD2 | 6.61E-01 | 4.47E-02 |  |  |  |
| LINC00327 | 7.98E-01 | 4.48E-02 |  |  |  |
| HCAR3 | 7.49E-01 | 4.58E-02 |  |  |  |
| ITPRIPL1 | 6.99E-01 | 4.60E-02 |  |  |  |
| MATK | 7.86E-01 | 4.61E-02 |  |  |  |
| ARHGAP22 | 5.75E-01 | 4.62E-02 |  |  |  |
| PTGDS | 9.19E-01 | 4.71E-02 |  |  |  |
| IRF8 | 7.89E-01 | 4.71E-02 |  |  |  |
| PER1 | 6.89E-01 | 4.74E-02 |  |  |  |
| C10orf54 | 6.46E-01 | 4.74E-02 |  |  |  |
| ZFP36L1 | 5.23E-01 | 4.75E-02 |  |  |  |
| TPTE | 9.90E-01 | 4.77E-02 |  |  |  |
| MAL | 9.72E-01 | 4.78E-02 |  |  |  |
| TRAV12-2 | 9.61E-01 | 4.78E-02 |  |  |  |
| KIRREL3 | 7.95E-01 | 4.78E-02 |  |  |  |
| CECR1 | 7.22E-01 | 4.79E-02 |  |  |  |
| ZBTB20-AS1 | 9.34E-01 | 4.83E-02 |  |  |  |
| CTC-459I6.1 | 7.53E-01 | 4.85E-02 |  |  |  |
| KLF4 | 7.51E-01 | 4.85E-02 |  |  |  |
| TNFAIP2 | 5.35E-01 | 4.85E-02 |  |  |  |
| AANAT | 7.81E-01 | 4.86E-02 |  |  |  |
| CCL4 | 8.11E-01 | 4.89E-02 |  |  |  |
| CCND2 | 6.18E-01 | 5.04E-02 |  |  |  |
| MMP9 | 8.80E-01 | 5.14E-02 |  |  |  |
| NUDT8 | 8.28E-01 | 5.14E-02 |  |  |  |
| RP4-639F20.1 | 7.07E-01 | 5.15E-02 |  |  |  |
| CYP17A1-AS1 | 8.11E-01 | 5.24E-02 |  |  |  |
| CSF2RB | 7.37E-01 | 5.24E-02 |  |  |  |
| CPLX2 | 9.24E-01 | 5.27E-02 |  |  |  |
| BTN2A2 | 5.09E-01 | 5.30E-02 |  |  |  |
| TRBV19 | 9.59E-01 | 5.31E-02 |  |  |  |
| MYO1F | 6.07E-01 | 5.31E-02 |  |  |  |
| RP11-23P13.6 | 8.73E-01 | 5.32E-02 |  |  |  |
| NCF1B | 6.96E-01 | 5.32E-02 |  |  |  |
| ST6GALNAC1 | 9.36E-01 | 5.33E-02 |  |  |  |
| TRAV19 | 9.74E-01 | 5.34E-02 |  |  |  |
| HES1 | 6.23E-01 | 5.34E-02 |  |  |  |
| ICAM3 | 6.01E-01 | 5.36E-02 |  |  |  |
| EGR2 | 8.05E-01 | 5.37E-02 |  |  |  |
| LINC00954 | 8.00E-01 | 5.44E-02 |  |  |  |
| GIMAP1 | 6.72E-01 | 5.44E-02 |  |  |  |
| CD300C | 6.13E-01 | 5.44E-02 |  |  |  |
| GUCY1A3 | 7.61E-01 | 5.44E-02 |  |  |  |
| RP11-290F5.1 | 9.48E-01 | 5.47E-02 |  |  |  |
| VAV1 | 7.05E-01 | 5.48E-02 |  |  |  |
| TRBV27 | 9.08E-01 | 5.55E-02 |  |  |  |
| CD200R1 | 7.60E-01 | 5.55E-02 |  |  |  |
| LINC00324 | 6.02E-01 | 5.55E-02 |  |  |  |
| LINC00582 | 8.13E-01 | 5.62E-02 |  |  |  |
| CYTIP | 8.12E-01 | 5.66E-02 |  |  |  |
| OCSTAMP | 9.42E-01 | 5.66E-02 |  |  |  |
| CYP17A1 | 7.59E-01 | 5.70E-02 |  |  |  |
| BEAN1 | 6.23E-01 | 5.70E-02 |  |  |  |
| TRAV9-2 | 9.52E-01 | 5.70E-02 |  |  |  |
| RAD21L1 | 9.31E-01 | 5.70E-02 |  |  |  |
| CYTH4 | 6.59E-01 | 5.70E-02 |  |  |  |
| DDX26B | 5.83E-01 | 5.70E-02 |  |  |  |
| NFATC1 | 5.85E-01 | 5.71E-02 |  |  |  |
| GRAP | 6.25E-01 | 5.73E-02 |  |  |  |
| SPSB4 | 8.37E-01 | 5.73E-02 |  |  |  |
| DNASE1L3 | 9.22E-01 | 5.75E-02 |  |  |  |
| CYP4Z2P | 9.42E-01 | 5.76E-02 |  |  |  |
| PELI2 | 6.71E-01 | 5.77E-02 |  |  |  |
| SELPLG | 6.15E-01 | 5.81E-02 |  |  |  |
| TMEM86A | 5.78E-01 | 5.81E-02 |  |  |  |
| BTN3A2 | 5.74E-01 | 5.81E-02 |  |  |  |
| RASD1 | 8.59E-01 | 5.82E-02 |  |  |  |
| ANKRD36BP2 | 8.83E-01 | 5.87E-02 |  |  |  |
| CYP4A11 | 8.35E-01 | 5.90E-02 |  |  |  |
| GPR65 | 5.91E-01 | 5.90E-02 |  |  |  |
| C5orf58 | 6.14E-01 | 5.93E-02 |  |  |  |
| CD1B | 9.49E-01 | 5.94E-02 |  |  |  |
| SLC16A11 | 9.11E-01 | 5.94E-02 |  |  |  |
| NEURL3 | 7.76E-01 | 5.94E-02 |  |  |  |
| RASGRF1 | 7.59E-01 | 5.94E-02 |  |  |  |
| TBX5 | 6.99E-01 | 5.94E-02 |  |  |  |
| HLA-DMB | 6.24E-01 | 5.94E-02 |  |  |  |
| SENCR | 6.70E-01 | 5.97E-02 |  |  |  |
| IGLV3-1 | 9.19E-01 | 5.99E-02 |  |  |  |
| RP5-884C9.2 | 9.42E-01 | 6.01E-02 |  |  |  |
| KCNK4 | 9.48E-01 | 6.01E-02 |  |  |  |
| APOBEC3H | 7.48E-01 | 6.12E-02 |  |  |  |
| DNMT3L | 9.41E-01 | 6.12E-02 |  |  |  |
| TRAV20 | 9.30E-01 | 6.23E-02 |  |  |  |
| AMICA1 | 7.79E-01 | 6.24E-02 |  |  |  |
| ACSL5 | 6.48E-01 | 6.24E-02 |  |  |  |
| MIR155HG | 7.72E-01 | 6.30E-02 |  |  |  |
| RGS16 | 5.47E-01 | 6.30E-02 |  |  |  |
| SAP25 | 5.05E-01 | 6.35E-02 |  |  |  |
| GJB6 | 9.25E-01 | 6.36E-02 |  |  |  |
| SIGLEC10 | 6.48E-01 | 6.38E-02 |  |  |  |
| BX255923.3 | 8.53E-01 | 6.38E-02 |  |  |  |
| LBH | 6.68E-01 | 6.40E-02 |  |  |  |
| TRBV29-1 | 8.87E-01 | 6.41E-02 |  |  |  |
| RASL10B | 8.31E-01 | 6.42E-02 |  |  |  |
| GDNF | 9.38E-01 | 6.42E-02 |  |  |  |
| MUSTN1 | 6.77E-01 | 6.42E-02 |  |  |  |
| CD226 | 6.55E-01 | 6.42E-02 |  |  |  |
| ARAP2 | 6.44E-01 | 6.42E-02 |  |  |  |
| GNG4 | 7.35E-01 | 6.47E-02 |  |  |  |
| RP11-16E12.2 | 7.84E-01 | 6.50E-02 |  |  |  |
| C14orf64 | 9.00E-01 | 6.52E-02 |  |  |  |
| MYO1G | 6.82E-01 | 6.56E-02 |  |  |  |
| ARID5A | 5.01E-01 | 6.56E-02 |  |  |  |
| CLEC17A | 9.36E-01 | 6.57E-02 |  |  |  |
| HLA-E | 5.40E-01 | 6.57E-02 |  |  |  |
| CD48 | 8.02E-01 | 6.59E-02 |  |  |  |
| CD28 | 8.55E-01 | 6.62E-02 |  |  |  |
| DLG2 | 7.63E-01 | 6.64E-02 |  |  |  |
| PPFIA2 | 7.79E-01 | 6.76E-02 |  |  |  |
| ARHGAP24 | 5.29E-01 | 6.76E-02 |  |  |  |
| BIN2 | 7.02E-01 | 6.82E-02 |  |  |  |
| GIMAP5 | 6.81E-01 | 6.92E-02 |  |  |  |
| S1PR4 | 8.66E-01 | 6.96E-02 |  |  |  |
| LRRC8C | 5.53E-01 | 6.97E-02 |  |  |  |
| SYTL3 | 5.02E-01 | 6.97E-02 |  |  |  |
| VPREB3 | 9.30E-01 | 6.97E-02 |  |  |  |
| SLAMF7 | 8.39E-01 | 6.97E-02 |  |  |  |
| PDK1 | 5.15E-01 | 6.97E-02 |  |  |  |
| HCLS1 | 6.30E-01 | 6.98E-02 |  |  |  |
| TPTE2 | 7.54E-01 | 7.01E-02 |  |  |  |
| SLC38A11 | 7.22E-01 | 7.01E-02 |  |  |  |
| GPR18 | 5.59E-01 | 7.10E-02 |  |  |  |
| RP11-81H14.2 | 9.06E-01 | 7.12E-02 |  |  |  |
| GBP2 | 6.26E-01 | 7.16E-02 |  |  |  |
| CTRC | 7.28E-01 | 7.16E-02 |  |  |  |
| IL10RA | 6.75E-01 | 7.16E-02 |  |  |  |
| HCG27 | 6.50E-01 | 7.17E-02 |  |  |  |
| IGHV2-26 | 9.21E-01 | 7.18E-02 |  |  |  |
| PARVG | 6.05E-01 | 7.20E-02 |  |  |  |
| TRBV5-1 | 8.59E-01 | 7.20E-02 |  |  |  |
| CDH23 | 5.70E-01 | 7.21E-02 |  |  |  |
| ZDHHC14 | 5.52E-01 | 7.22E-02 |  |  |  |
| CD101 | 5.52E-01 | 7.26E-02 |  |  |  |
| TRBV2 | 8.72E-01 | 7.28E-02 |  |  |  |
| TRAV8-1 | 9.13E-01 | 7.36E-02 |  |  |  |
| USP44 | 8.59E-01 | 7.46E-02 |  |  |  |
| RP11-439L8.3 | 8.97E-01 | 7.46E-02 |  |  |  |
| CASP10 | 5.33E-01 | 7.67E-02 |  |  |  |
| GIMAP8 | 5.74E-01 | 7.68E-02 |  |  |  |
| RP11-409K20.6 | 5.70E-01 | 7.68E-02 |  |  |  |
| AC104698.1 | 6.34E-01 | 7.71E-02 |  |  |  |
| GTSF1 | 8.01E-01 | 7.72E-02 |  |  |  |
| KIAA0226L | 6.62E-01 | 7.72E-02 |  |  |  |
| CEACAM21 | 6.87E-01 | 7.75E-02 |  |  |  |
| MANSC4 | 6.33E-01 | 7.75E-02 |  |  |  |
| FLRT2 | 7.55E-01 | 7.79E-02 |  |  |  |
| LIX1L | 5.48E-01 | 7.79E-02 |  |  |  |
| TRBV9 | 8.92E-01 | 7.81E-02 |  |  |  |
| TRAV17 | 9.02E-01 | 7.83E-02 |  |  |  |
| HLA-F-AS1 | 5.51E-01 | 7.83E-02 |  |  |  |
| IL15 | 5.50E-01 | 7.85E-02 |  |  |  |
| GNLY | 7.94E-01 | 7.87E-02 |  |  |  |
| ABI3 | 5.23E-01 | 7.90E-02 |  |  |  |
| GIMAP7 | 6.68E-01 | 7.96E-02 |  |  |  |
| CACNA2D3 | 6.33E-01 | 7.96E-02 |  |  |  |
| CYP2U1 | 5.22E-01 | 7.96E-02 |  |  |  |
| MPEG1 | 6.96E-01 | 7.97E-02 |  |  |  |
| PRR5L | 5.02E-01 | 7.97E-02 |  |  |  |
| CCRL2 | 5.65E-01 | 8.00E-02 |  |  |  |
| RGN | 8.16E-01 | 8.01E-02 |  |  |  |
| KIAA1644 | 7.65E-01 | 8.02E-02 |  |  |  |
| C3 | 6.05E-01 | 8.03E-02 |  |  |  |
| RAB37 | 5.23E-01 | 8.07E-02 |  |  |  |
| RUNX3 | 7.34E-01 | 8.21E-02 |  |  |  |
| TMEM221 | 6.94E-01 | 8.41E-02 |  |  |  |
| SLC2A12 | 5.56E-01 | 8.42E-02 |  |  |  |
| NPHS1 | 8.85E-01 | 8.47E-02 |  |  |  |
| IGHGP | 7.14E-01 | 8.47E-02 |  |  |  |
| TMEM132E | 7.99E-01 | 8.52E-02 |  |  |  |
| GALNT12 | 6.96E-01 | 8.52E-02 |  |  |  |
| SLFN12L | 8.16E-01 | 8.56E-02 |  |  |  |
| LCN10 | 8.64E-01 | 8.60E-02 |  |  |  |
| ATP1B2 | 6.65E-01 | 8.62E-02 |  |  |  |
| CCL22 | 7.65E-01 | 8.63E-02 |  |  |  |
| KLRG1 | 6.84E-01 | 8.66E-02 |  |  |  |
| RP11-265P11.2 | 8.88E-01 | 8.70E-02 |  |  |  |
| IGHM | 8.34E-01 | 8.70E-02 |  |  |  |
| CLECL1 | 8.04E-01 | 8.70E-02 |  |  |  |
| GPR171 | 8.19E-01 | 8.74E-02 |  |  |  |
| NTNG2 | 6.97E-01 | 8.77E-02 |  |  |  |
| CLU | 6.62E-01 | 8.77E-02 |  |  |  |
| RPL13AP17 | 8.92E-01 | 8.82E-02 |  |  |  |
| RP11-439L18.3 | 6.76E-01 | 8.83E-02 |  |  |  |
| LINC00930 | 7.42E-01 | 8.83E-02 |  |  |  |
| HLA-DRA | 5.21E-01 | 8.88E-02 |  |  |  |
| SASH3 | 7.14E-01 | 8.90E-02 |  |  |  |
| LTF | 8.89E-01 | 9.00E-02 |  |  |  |
| TNFSF10 | 5.98E-01 | 9.00E-02 |  |  |  |
| RGS13 | 8.32E-01 | 9.01E-02 |  |  |  |
| TRBV5-6 | 8.85E-01 | 9.04E-02 |  |  |  |
| ITGA4 | 7.12E-01 | 9.20E-02 |  |  |  |
| EBI3 | 6.82E-01 | 9.20E-02 |  |  |  |
| SGPP1 | 5.41E-01 | 9.20E-02 |  |  |  |
| TGM1 | 5.00E-01 | 9.27E-02 |  |  |  |
| HGF | 5.64E-01 | 9.35E-02 |  |  |  |
| DMRTC1B | 7.51E-01 | 9.43E-02 |  |  |  |
| PTPRD | 7.05E-01 | 9.43E-02 |  |  |  |
| RAC2 | 6.96E-01 | 9.43E-02 |  |  |  |
| SFMBT2 | 5.63E-01 | 9.49E-02 |  |  |  |
| NLRC5 | 6.02E-01 | 9.51E-02 |  |  |  |
| ZFPM2 | 5.86E-01 | 9.51E-02 |  |  |  |
| AIM2 | 8.34E-01 | 9.52E-02 |  |  |  |
| FGL2 | 6.10E-01 | 9.52E-02 |  |  |  |
| ANKRD33B | 8.65E-01 | 9.58E-02 |  |  |  |
| CTD-2370N5.3 | 6.44E-01 | 9.64E-02 |  |  |  |
| MUC19 | 8.53E-01 | 9.65E-02 |  |  |  |
| XXbac-BPG254F23.6 | 7.33E-01 | 9.65E-02 |  |  |  |
| FAM159A | 7.16E-01 | 9.67E-02 |  |  |  |
| CERKL | 5.39E-01 | 9.68E-02 |  |  |  |
| TRBV10-2 | 8.70E-01 | 9.71E-02 |  |  |  |
| GVINP1 | 7.60E-01 | 9.73E-02 |  |  |  |
| CNGA3 | 8.71E-01 | 9.77E-02 |  |  |  |
| HILS1 | 8.03E-01 | 9.77E-02 |  |  |  |
| ADAMDEC1 | 8.47E-01 | 9.78E-02 |  |  |  |
| BPIFA4P | 8.39E-01 | 9.82E-02 |  |  |  |
| IDO1 | 8.40E-01 | 9.83E-02 |  |  |  |
| TMC5 | 7.99E-01 | 9.85E-02 |  |  |  |
| TRAV24 | 8.63E-01 | 9.90E-02 |  |  |  |
| AP003774.4 | 5.70E-01 | 9.93E-02 |  |  |  |
| ATP13A4 | 8.51E-01 | 9.93E-02 |  |  |  |
| CRTAM | 7.61E-01 | 9.97E-02 |  |  |  |
| C9orf139 | 6.18E-01 | 9.98E-02 |  |  |  |
| **Down-regulated genes** | | | | | |
| GREB1 | -2.03E+00 | 4.22E-10 | CENPU | -1.10E+00 | 1.04E-04 |
| NPY1R | -2.32E+00 | 3.82E-09 | CENPE | -1.34E+00 | 3.33E-04 |
| TMEM26 | -1.99E+00 | 4.09E-09 | TOP2A | -1.53E+00 | 3.33E-04 |
| C5AR2 | -1.53E+00 | 6.39E-09 | STMN1 | -9.31E-01 | 1.51E-03 |
| C6orf141 | -2.05E+00 | 3.88E-08 | DEPDC1B | -1.35E+00 | 4.84E-03 |
| FMN1 | -1.23E+00 | 2.50E-07 | HJURP | -1.35E+00 | 5.05E-03 |
| MAG | -1.99E+00 | 6.42E-07 | DLGAP5 | -1.33E+00 | 5.24E-03 |
| PGR | -1.92E+00 | 1.13E-06 | CDC6 | -1.28E+00 | 6.06E-03 |
| GFRA1 | -1.79E+00 | 1.60E-06 | TTK | -1.17E+00 | 6.53E-03 |
| STC1 | -1.76E+00 | 3.25E-06 | CCNB1 | -1.11E+00 | 7.57E-03 |
| FAM196A | -1.86E+00 | 6.72E-06 | NDC80 | -1.19E+00 | 7.57E-03 |
| LINC01016 | -1.83E+00 | 8.63E-06 | KIAA0101 | -1.23E+00 | 7.57E-03 |
| RIMS4 | -1.83E+00 | 8.63E-06 | NCAPG | -1.04E+00 | 8.84E-03 |
| HBB | -1.73E+00 | 3.23E-05 | BUB1B | -1.16E+00 | 8.96E-03 |
| ZNF587B | -6.29E-01 | 4.20E-05 | LMNB2 | -6.71E-01 | 1.02E-02 |
| CTD-2583A14.10 | -6.32E-01 | 4.20E-05 | KIF20A | -1.19E+00 | 1.02E-02 |
| IL20 | -1.77E+00 | 4.20E-05 | CDC20 | -1.25E+00 | 1.02E-02 |
| LPPR3 | -1.65E+00 | 8.03E-05 | CCT5 | -5.17E-01 | 1.17E-02 |
| LINC00160 | -1.58E+00 | 9.70E-05 | CDCA8 | -9.66E-01 | 1.17E-02 |
| RBM24 | -1.60E+00 | 9.70E-05 | PRC1 | -1.10E+00 | 1.17E-02 |
| NOS1AP | -1.55E+00 | 1.25E-04 | CLSPN | -1.11E+00 | 1.17E-02 |
| HBA1 | -1.61E+00 | 1.25E-04 | CDC25C | -1.13E+00 | 1.17E-02 |
| THSD4 | -1.14E+00 | 1.48E-04 | NUSAP1 | -1.16E+00 | 1.17E-02 |
| CKS2 | -8.94E-01 | 1.70E-04 | SKA3 | -1.21E+00 | 1.17E-02 |
| PPFIA4 | -1.50E+00 | 2.01E-04 | KIF2C | -1.23E+00 | 1.17E-02 |
| HBA2 | -1.57E+00 | 2.01E-04 | MELK | -1.27E+00 | 1.17E-02 |
| AZU1 | -1.59E+00 | 2.01E-04 | SKA1 | -1.12E+00 | 1.20E-02 |
| GRPR | -1.63E+00 | 2.01E-04 | CCNA2 | -9.55E-01 | 1.25E-02 |
| LINC00238 | -1.63E+00 | 2.01E-04 | PRC1-AS1 | -1.09E+00 | 1.27E-02 |
| TPBG | -9.42E-01 | 2.03E-04 | C5AR2 | -1.02E+00 | 1.31E-02 |
| C1orf226 | -1.44E+00 | 2.98E-04 | CIT | -1.13E+00 | 1.31E-02 |
| RP11-565P22.6 | -1.40E+00 | 3.47E-04 | LAPTM4B | -1.20E+00 | 1.31E-02 |
| C1orf173 | -1.58E+00 | 4.51E-04 | KIF4A | -1.24E+00 | 1.31E-02 |
| CCNB1 | -9.27E-01 | 4.85E-04 | NEIL3 | -1.29E+00 | 1.31E-02 |
| SUSD3 | -1.28E+00 | 4.85E-04 | MYBL2 | -1.32E+00 | 1.31E-02 |
| CDC25C | -1.11E+00 | 5.97E-04 | TEX14 | -1.35E+00 | 1.31E-02 |
| MREG | -8.33E-01 | 6.16E-04 | KIFC1 | -1.02E+00 | 1.36E-02 |
| ADORA1 | -1.35E+00 | 6.83E-04 | SPAG5 | -1.07E+00 | 1.36E-02 |
| PDZK1 | -1.51E+00 | 7.87E-04 | BLM | -1.04E+00 | 1.45E-02 |
| ZNF587 | -9.20E-01 | 8.24E-04 | H2AFZ | -6.22E-01 | 1.46E-02 |
| MSMB | -1.44E+00 | 8.46E-04 | CCNB2 | -1.18E+00 | 1.46E-02 |
| PRC1 | -1.03E+00 | 8.78E-04 | PLK4 | -9.89E-01 | 1.49E-02 |
| IGSF1 | -1.51E+00 | 9.03E-04 | MAD2L1 | -1.01E+00 | 1.51E-02 |
| PRC1-AS1 | -1.03E+00 | 9.04E-04 | BUB1 | -1.06E+00 | 1.51E-02 |
| IL6ST | -8.59E-01 | 1.16E-03 | CDKN3 | -1.19E+00 | 1.51E-02 |
| RBM20 | -1.44E+00 | 1.17E-03 | IL19 | -1.31E+00 | 1.52E-02 |
| IGF1R | -1.01E+00 | 1.26E-03 | AURKB | -1.27E+00 | 1.55E-02 |
| ZNF814 | -7.98E-01 | 1.34E-03 | CKS2 | -1.04E+00 | 1.56E-02 |
| C1orf111 | -1.42E+00 | 1.40E-03 | PTTG1 | -1.20E+00 | 1.79E-02 |
| FAM64A | -9.59E-01 | 1.41E-03 | TRIP13 | -1.03E+00 | 1.86E-02 |
| CT62 | -1.16E+00 | 1.41E-03 | LMNB1 | -1.01E+00 | 1.87E-02 |
| ELOVL2 | -1.41E+00 | 1.41E-03 | E2F7 | -1.13E+00 | 1.99E-02 |
| MYBL1 | -1.30E+00 | 1.43E-03 | PLK1 | -1.15E+00 | 1.99E-02 |
| TEX14 | -1.39E+00 | 1.47E-03 | TACC3 | -9.73E-01 | 2.15E-02 |
| CTD-2033D15.1 | -9.24E-01 | 1.52E-03 | KPNA2 | -9.11E-01 | 2.23E-02 |
| TUBA3D | -1.26E+00 | 1.62E-03 | ESPL1 | -1.11E+00 | 2.23E-02 |
| GINS2 | -8.87E-01 | 1.67E-03 | FAM72D | -1.06E+00 | 2.27E-02 |
| UBE2C | -1.18E+00 | 1.82E-03 | UBE2C | -1.23E+00 | 2.30E-02 |
| MAPT-AS1 | -1.30E+00 | 1.84E-03 | TPX2 | -1.14E+00 | 2.31E-02 |
| RP6-74O6.2 | -1.42E+00 | 1.84E-03 | KIF11 | -9.54E-01 | 2.44E-02 |
| SCUBE1 | -1.37E+00 | 1.88E-03 | CDCA5 | -1.12E+00 | 2.48E-02 |
| PDZK1P1 | -1.40E+00 | 2.06E-03 | DTYMK | -6.50E-01 | 2.61E-02 |
| TRIP13 | -9.44E-01 | 2.26E-03 | RACGAP1 | -8.99E-01 | 2.66E-02 |
| SUB1 | -7.59E-01 | 2.30E-03 | FEN1 | -7.02E-01 | 2.88E-02 |
| PDZK1P2 | -1.39E+00 | 2.33E-03 | TROAP | -1.13E+00 | 2.88E-02 |
| TERT | -1.27E+00 | 2.45E-03 | DEPDC1 | -1.11E+00 | 3.01E-02 |
| CYP2A7 | -1.36E+00 | 2.55E-03 | NT5DC4 | -1.13E+00 | 3.01E-02 |
| EGLN2 | -7.55E-01 | 2.62E-03 | GGH | -1.18E+00 | 3.01E-02 |
| UBE2S | -7.85E-01 | 2.96E-03 | DIAPH3 | -1.11E+00 | 3.12E-02 |
| DCAF10 | -8.83E-01 | 3.18E-03 | SGOL2 | -7.13E-01 | 3.20E-02 |
| PLAC1 | -1.21E+00 | 3.25E-03 | CENPK | -1.02E+00 | 3.32E-02 |
| PAICS | -5.25E-01 | 3.34E-03 | FAM64A | -1.11E+00 | 3.32E-02 |
| DYNLL1-AS1 | -5.50E-01 | 3.34E-03 | MZT2A | -7.81E-01 | 3.39E-02 |
| KNSTRN | -6.31E-01 | 3.34E-03 | TYMS | -6.88E-01 | 3.41E-02 |
| C9orf89 | -6.43E-01 | 3.34E-03 | EPS15L1 | -5.86E-01 | 3.55E-02 |
| TTLL12 | -9.47E-01 | 3.34E-03 | EGLN2 | -9.21E-01 | 3.94E-02 |
| NUF2 | -9.03E-01 | 3.51E-03 | GREB1 | -1.19E+00 | 4.02E-02 |
| RAD51 | -8.77E-01 | 3.60E-03 | TP73 | -8.99E-01 | 4.12E-02 |
| PMAIP1 | -1.05E+00 | 3.72E-03 | SGOL1 | -1.00E+00 | 4.63E-02 |
| NRIP1 | -6.02E-01 | 3.92E-03 | CENPF | -1.07E+00 | 4.65E-02 |
| MAPT | -1.12E+00 | 3.95E-03 | BIRC5 | -1.15E+00 | 4.78E-02 |
| ADCY1 | -1.29E+00 | 3.95E-03 | NUF2 | -9.55E-01 | 4.79E-02 |
| UCK2 | -5.11E-01 | 4.02E-03 | FAM83D | -1.12E+00 | 4.79E-02 |
| CHST8 | -1.33E+00 | 4.02E-03 | TERT | -1.19E+00 | 4.81E-02 |
| TROAP | -1.04E+00 | 4.28E-03 | ARHGAP11A | -9.36E-01 | 4.90E-02 |
| STH | -1.15E+00 | 4.28E-03 | OIP5 | -9.75E-01 | 4.93E-02 |
| TMC3 | -1.26E+00 | 4.65E-03 | KIF23 | -9.97E-01 | 4.95E-02 |
| CCNF | -6.39E-01 | 4.66E-03 | PIF1 | -1.05E+00 | 4.95E-02 |
| KPNA2 | -7.01E-01 | 4.66E-03 | ASF1B | -1.06E+00 | 5.24E-02 |
| RACGAP1 | -7.86E-01 | 4.66E-03 | SMPD4 | -5.57E-01 | 5.44E-02 |
| RP4-539M6.19 | -9.90E-01 | 4.66E-03 | AURKA | -9.37E-01 | 5.72E-02 |
| RP11-761I4.3 | -1.26E+00 | 4.69E-03 | RAB4B-EGLN2 | -8.19E-01 | 6.26E-02 |
| CELSR2 | -1.03E+00 | 4.73E-03 | HMMR | -1.02E+00 | 6.26E-02 |
| HSDL2 | -5.50E-01 | 4.75E-03 | KIF18B | -1.04E+00 | 6.35E-02 |
| C9orf147 | -5.41E-01 | 5.10E-03 | C9orf89 | -7.82E-01 | 6.55E-02 |
| CDC20 | -9.99E-01 | 5.23E-03 | HMGB2 | -8.66E-01 | 6.55E-02 |
| CLSTN2 | -1.12E+00 | 5.25E-03 | NCAPH | -9.92E-01 | 6.61E-02 |
| RNF223 | -1.09E+00 | 5.26E-03 | GTSE1 | -1.06E+00 | 6.61E-02 |
| UBE2T | -9.13E-01 | 5.35E-03 | BRCA1 | -7.95E-01 | 6.62E-02 |
| CENPU | -7.45E-01 | 5.44E-03 | SHCBP1 | -1.04E+00 | 7.16E-02 |
| TAT | -1.29E+00 | 5.46E-03 | CEP55 | -1.04E+00 | 7.16E-02 |
| ARHGAP11A | -9.97E-01 | 5.87E-03 | WDR62 | -9.30E-01 | 7.19E-02 |
| TOP2A | -1.11E+00 | 5.87E-03 | CKAP2L | -9.98E-01 | 7.27E-02 |
| SGK3 | -1.04E+00 | 5.88E-03 | RDM1 | -1.11E+00 | 7.27E-02 |
| OIP5 | -8.51E-01 | 6.04E-03 | HN1 | -5.22E-01 | 7.47E-02 |
| THBS1 | -7.56E-01 | 6.09E-03 | TIMELESS | -6.17E-01 | 9.55E-02 |
| UGCG | -9.26E-01 | 6.59E-03 | TPBG | -8.23E-01 | 9.57E-02 |
| NT5DC4 | -9.74E-01 | 6.92E-03 | CELSR2 | -9.80E-01 | 9.57E-02 |
| CENPM | -9.35E-01 | 6.93E-03 | IQGAP3 | -1.03E+00 | 9.57E-02 |
| AURKA | -9.35E-01 | 7.02E-03 | PREX1 | -8.06E-01 | 9.77E-02 |
| RABEP1 | -6.04E-01 | 7.09E-03 |  |  |  |
| ZNF92 | -9.89E-01 | 7.26E-03 |  |  |  |
| NKAIN1 | -1.26E+00 | 7.28E-03 |  |  |  |
| ESCO2 | -9.55E-01 | 7.29E-03 |  |  |  |
| SERPINA5 | -1.24E+00 | 7.88E-03 |  |  |  |
| CENPH | -5.99E-01 | 7.94E-03 |  |  |  |
| CKAP2L | -9.99E-01 | 8.12E-03 |  |  |  |
| TRH | -1.24E+00 | 8.12E-03 |  |  |  |
| WDR62 | -8.87E-01 | 8.35E-03 |  |  |  |
| FGFR3 | -1.10E+00 | 8.48E-03 |  |  |  |
| PBK | -1.08E+00 | 8.50E-03 |  |  |  |
| HMGB2 | -6.26E-01 | 8.90E-03 |  |  |  |
| ELOVL2-AS1 | -1.23E+00 | 8.97E-03 |  |  |  |
| CDK1 | -9.09E-01 | 9.01E-03 |  |  |  |
| NUP88 | -5.39E-01 | 9.04E-03 |  |  |  |
| RP11-65J21.1 | -1.22E+00 | 9.04E-03 |  |  |  |
| LDLRAD3 | -9.22E-01 | 9.25E-03 |  |  |  |
| SEC14L2 | -1.00E+00 | 9.25E-03 |  |  |  |
| ADCY9 | -7.16E-01 | 9.47E-03 |  |  |  |
| C16orf59 | -7.57E-01 | 9.59E-03 |  |  |  |
| LIN28A | -1.20E+00 | 9.59E-03 |  |  |  |
| SGOL2 | -6.71E-01 | 9.62E-03 |  |  |  |
| CAP2 | -9.03E-01 | 9.78E-03 |  |  |  |
| ZNF367 | -8.38E-01 | 9.86E-03 |  |  |  |
| ASCL1 | -1.16E+00 | 9.94E-03 |  |  |  |
| CCNB2 | -9.61E-01 | 1.02E-02 |  |  |  |
| POC1A | -7.01E-01 | 1.03E-02 |  |  |  |
| SPPL2C | -1.19E+00 | 1.05E-02 |  |  |  |
| NEK2 | -9.92E-01 | 1.05E-02 |  |  |  |
| RAD51AP1 | -7.47E-01 | 1.06E-02 |  |  |  |
| KCNK6 | -8.14E-01 | 1.06E-02 |  |  |  |
| SERPINA12 | -1.20E+00 | 1.06E-02 |  |  |  |
| KCNK15 | -1.01E+00 | 1.10E-02 |  |  |  |
| C8orf44-SGK3 | -9.35E-01 | 1.10E-02 |  |  |  |
| NLN | -5.38E-01 | 1.10E-02 |  |  |  |
| IRS1 | -9.48E-01 | 1.10E-02 |  |  |  |
| SCNN1G | -1.20E+00 | 1.10E-02 |  |  |  |
| CDC6 | -9.46E-01 | 1.13E-02 |  |  |  |
| RAB4B-EGLN2 | -6.03E-01 | 1.15E-02 |  |  |  |
| SLC25A24 | -5.46E-01 | 1.20E-02 |  |  |  |
| KIF23 | -7.83E-01 | 1.20E-02 |  |  |  |
| BSN | -1.04E+00 | 1.22E-02 |  |  |  |
| NEIL3 | -9.77E-01 | 1.22E-02 |  |  |  |
| SSX2IP | -5.92E-01 | 1.25E-02 |  |  |  |
| PARD6B | -9.71E-01 | 1.25E-02 |  |  |  |
| KIF18A | -9.01E-01 | 1.29E-02 |  |  |  |
| ATAD2 | -7.96E-01 | 1.33E-02 |  |  |  |
| TPX2 | -9.39E-01 | 1.36E-02 |  |  |  |
| LETM1 | -5.03E-01 | 1.39E-02 |  |  |  |
| ZNF552 | -7.51E-01 | 1.39E-02 |  |  |  |
| SAPCD2 | -7.56E-01 | 1.43E-02 |  |  |  |
| DSCC1 | -6.19E-01 | 1.43E-02 |  |  |  |
| SLC29A3 | -6.33E-01 | 1.46E-02 |  |  |  |
| NPY5R | -1.16E+00 | 1.46E-02 |  |  |  |
| MCM10 | -9.47E-01 | 1.49E-02 |  |  |  |
| BUB1B | -8.71E-01 | 1.58E-02 |  |  |  |
| LAMA3 | -9.88E-01 | 1.58E-02 |  |  |  |
| KCNH6 | -1.15E+00 | 1.58E-02 |  |  |  |
| AMIGO2 | -9.58E-01 | 1.59E-02 |  |  |  |
| PGLYRP2 | -1.13E+00 | 1.59E-02 |  |  |  |
| NUSAP1 | -8.77E-01 | 1.60E-02 |  |  |  |
| CYP2A13 | -1.14E+00 | 1.60E-02 |  |  |  |
| RAD54B | -6.44E-01 | 1.63E-02 |  |  |  |
| RERG | -8.27E-01 | 1.75E-02 |  |  |  |
| GDAP1 | -1.07E+00 | 1.75E-02 |  |  |  |
| CDC45 | -9.26E-01 | 1.77E-02 |  |  |  |
| MS4A8 | -1.13E+00 | 1.77E-02 |  |  |  |
| FSBP | -6.27E-01 | 1.80E-02 |  |  |  |
| RBBP8 | -7.59E-01 | 1.80E-02 |  |  |  |
| MAD2L1 | -7.35E-01 | 1.82E-02 |  |  |  |
| NXNL2 | -1.01E+00 | 1.82E-02 |  |  |  |
| KIF20A | -8.85E-01 | 1.84E-02 |  |  |  |
| E2F1 | -8.36E-01 | 1.84E-02 |  |  |  |
| PLK1 | -7.91E-01 | 1.94E-02 |  |  |  |
| FSIP1 | -1.08E+00 | 1.98E-02 |  |  |  |
| CIT | -8.04E-01 | 1.98E-02 |  |  |  |
| S100A8 | -1.08E+00 | 2.04E-02 |  |  |  |
| CCDC150 | -6.07E-01 | 2.05E-02 |  |  |  |
| GNG13 | -9.10E-01 | 2.10E-02 |  |  |  |
| MIR17HG | -5.41E-01 | 2.16E-02 |  |  |  |
| MBOAT1 | -5.81E-01 | 2.16E-02 |  |  |  |
| BRCA1 | -6.95E-01 | 2.17E-02 |  |  |  |
| SLC30A1 | -5.28E-01 | 2.23E-02 |  |  |  |
| CHPT1 | -6.96E-01 | 2.23E-02 |  |  |  |
| GRIK3 | -1.10E+00 | 2.23E-02 |  |  |  |
| SFXN2 | -6.36E-01 | 2.28E-02 |  |  |  |
| TTC26 | -5.26E-01 | 2.28E-02 |  |  |  |
| RAMP1 | -1.02E+00 | 2.31E-02 |  |  |  |
| SCNN1B | -1.06E+00 | 2.34E-02 |  |  |  |
| NAT16 | -1.07E+00 | 2.35E-02 |  |  |  |
| ZNF586 | -6.59E-01 | 2.36E-02 |  |  |  |
| TUBA3E | -1.07E+00 | 2.42E-02 |  |  |  |
| SIAH2 | -7.68E-01 | 2.44E-02 |  |  |  |
| KIF11 | -7.60E-01 | 2.45E-02 |  |  |  |
| PTTG1 | -8.29E-01 | 2.45E-02 |  |  |  |
| NDC80 | -8.08E-01 | 2.47E-02 |  |  |  |
| DOCK1 | -6.52E-01 | 2.50E-02 |  |  |  |
| CST4 | -1.07E+00 | 2.52E-02 |  |  |  |
| KIF4A | -8.97E-01 | 2.60E-02 |  |  |  |
| CXCL5 | -9.96E-01 | 2.64E-02 |  |  |  |
| PAH | -1.08E+00 | 2.65E-02 |  |  |  |
| RAD21 | -5.04E-01 | 2.71E-02 |  |  |  |
| HSD17B7 | -5.54E-01 | 2.79E-02 |  |  |  |
| KIF2C | -8.27E-01 | 2.81E-02 |  |  |  |
| CYP2G1P | -1.04E+00 | 2.83E-02 |  |  |  |
| GINS1 | -7.25E-01 | 2.85E-02 |  |  |  |
| AC104057.1 | -1.06E+00 | 2.87E-02 |  |  |  |
| LMNB1 | -6.97E-01 | 2.90E-02 |  |  |  |
| GINS4 | -7.74E-01 | 2.90E-02 |  |  |  |
| NME1 | -5.60E-01 | 2.90E-02 |  |  |  |
| ORC6 | -7.12E-01 | 2.91E-02 |  |  |  |
| ADAMTS15 | -9.52E-01 | 2.91E-02 |  |  |  |
| GRIK4 | -9.53E-01 | 2.97E-02 |  |  |  |
| NCAPG | -7.42E-01 | 3.04E-02 |  |  |  |
| NCAPH | -7.83E-01 | 3.09E-02 |  |  |  |
| EREG | -1.05E+00 | 3.14E-02 |  |  |  |
| E2F8 | -9.28E-01 | 3.16E-02 |  |  |  |
| ATP13A2 | -5.36E-01 | 3.17E-02 |  |  |  |
| PFKFB3 | -7.25E-01 | 3.21E-02 |  |  |  |
| CTA-392E5.1 | -1.04E+00 | 3.22E-02 |  |  |  |
| KIF18B | -8.55E-01 | 3.26E-02 |  |  |  |
| PPM1E | -1.05E+00 | 3.26E-02 |  |  |  |
| SERPINA3 | -9.68E-01 | 3.35E-02 |  |  |  |
| QDPR | -7.39E-01 | 3.43E-02 |  |  |  |
| CDCA8 | -6.61E-01 | 3.43E-02 |  |  |  |
| NEURL1 | -1.02E+00 | 3.44E-02 |  |  |  |
| ANKRD50 | -6.69E-01 | 3.45E-02 |  |  |  |
| DPY19L2P1 | -9.95E-01 | 3.45E-02 |  |  |  |
| PREX1 | -7.54E-01 | 3.56E-02 |  |  |  |
| CUEDC1 | -6.07E-01 | 3.56E-02 |  |  |  |
| TMEM145 | -1.01E+00 | 3.74E-02 |  |  |  |
| KIAA0101 | -7.81E-01 | 3.77E-02 |  |  |  |
| POLE2 | -5.76E-01 | 3.80E-02 |  |  |  |
| CCNA2 | -5.75E-01 | 3.82E-02 |  |  |  |
| HJURP | -8.28E-01 | 3.85E-02 |  |  |  |
| HMMR | -8.45E-01 | 3.87E-02 |  |  |  |
| SKA1 | -7.47E-01 | 3.90E-02 |  |  |  |
| DEPDC1 | -8.68E-01 | 3.96E-02 |  |  |  |
| RP11-286B14.1 | -9.92E-01 | 3.97E-02 |  |  |  |
| FOXM1 | -5.41E-01 | 4.03E-02 |  |  |  |
| BIRC5 | -8.88E-01 | 4.06E-02 |  |  |  |
| SLC6A4 | -1.00E+00 | 4.06E-02 |  |  |  |
| HIST1H4A | -1.01E+00 | 4.07E-02 |  |  |  |
| SERPINA4 | -1.01E+00 | 4.07E-02 |  |  |  |
| ESPL1 | -8.72E-01 | 4.07E-02 |  |  |  |
| RNF215 | -6.53E-01 | 4.08E-02 |  |  |  |
| RAD54L | -5.88E-01 | 4.17E-02 |  |  |  |
| ARHGAP11B | -7.18E-01 | 4.30E-02 |  |  |  |
| RECQL4 | -6.80E-01 | 4.33E-02 |  |  |  |
| ZWINT | -6.99E-01 | 4.36E-02 |  |  |  |
| MTFP1 | -5.23E-01 | 4.42E-02 |  |  |  |
| CTB-167G5.5 | -9.99E-01 | 4.45E-02 |  |  |  |
| RP11-563J2.2 | -7.46E-01 | 4.56E-02 |  |  |  |
| CTSV | -7.47E-01 | 4.58E-02 |  |  |  |
| CENPE | -7.73E-01 | 4.62E-02 |  |  |  |
| KIAA1524 | -6.71E-01 | 4.62E-02 |  |  |  |
| ECT2 | -6.17E-01 | 4.71E-02 |  |  |  |
| INSM1 | -9.77E-01 | 4.75E-02 |  |  |  |
| MELK | -8.07E-01 | 4.77E-02 |  |  |  |
| SYTL4 | -5.91E-01 | 4.78E-02 |  |  |  |
| CENPF | -7.63E-01 | 4.78E-02 |  |  |  |
| TUBB1 | -7.51E-01 | 4.78E-02 |  |  |  |
| SLC19A2 | -5.80E-01 | 4.83E-02 |  |  |  |
| CENPK | -6.80E-01 | 4.83E-02 |  |  |  |
| DCAF13 | -5.10E-01 | 4.84E-02 |  |  |  |
| MIR3973 | -8.82E-01 | 4.85E-02 |  |  |  |
| PARPBP | -6.37E-01 | 4.89E-02 |  |  |  |
| AGR2 | -8.15E-01 | 4.95E-02 |  |  |  |
| PMCH | -6.21E-01 | 5.01E-02 |  |  |  |
| TFRC | -6.06E-01 | 5.16E-02 |  |  |  |
| DIAPH3 | -7.79E-01 | 5.21E-02 |  |  |  |
| ATP6V1B1 | -9.45E-01 | 5.28E-02 |  |  |  |
| RAMP3 | -8.38E-01 | 5.33E-02 |  |  |  |
| GPR26 | -9.61E-01 | 5.37E-02 |  |  |  |
| CTD-2510F5.6 | -5.44E-01 | 5.46E-02 |  |  |  |
| RDH16 | -8.90E-01 | 5.48E-02 |  |  |  |
| CYP2F1 | -8.71E-01 | 5.50E-02 |  |  |  |
| ISOC1 | -6.72E-01 | 5.71E-02 |  |  |  |
| FAM214A | -5.13E-01 | 5.73E-02 |  |  |  |
| NEURL1B | -5.42E-01 | 5.74E-02 |  |  |  |
| KDM4B | -5.81E-01 | 5.75E-02 |  |  |  |
| PPBP | -8.97E-01 | 5.77E-02 |  |  |  |
| ORC1 | -6.99E-01 | 5.81E-02 |  |  |  |
| CDCA5 | -7.72E-01 | 5.81E-02 |  |  |  |
| IQGAP3 | -8.27E-01 | 5.81E-02 |  |  |  |
| SVOP | -9.20E-01 | 5.81E-02 |  |  |  |
| RP11-95P2.3 | -9.43E-01 | 5.81E-02 |  |  |  |
| CEP55 | -7.91E-01 | 5.92E-02 |  |  |  |
| GLA | -5.83E-01 | 5.94E-02 |  |  |  |
| AXDND1 | -8.36E-01 | 5.94E-02 |  |  |  |
| CCDC117 | -6.76E-01 | 5.96E-02 |  |  |  |
| DOK7 | -9.32E-01 | 5.99E-02 |  |  |  |
| SSTR2 | -8.48E-01 | 6.11E-02 |  |  |  |
| CYP2A6 | -9.37E-01 | 6.12E-02 |  |  |  |
| ALS2CR12 | -7.69E-01 | 6.17E-02 |  |  |  |
| FAM65C | -6.66E-01 | 6.18E-02 |  |  |  |
| HDAC11 | -5.46E-01 | 6.23E-02 |  |  |  |
| BUB1 | -7.40E-01 | 6.24E-02 |  |  |  |
| RGS22 | -9.15E-01 | 6.30E-02 |  |  |  |
| LAPTM4B | -7.00E-01 | 6.34E-02 |  |  |  |
| PRR11 | -7.63E-01 | 6.36E-02 |  |  |  |
| CHRNB2 | -9.37E-01 | 6.37E-02 |  |  |  |
| PSRC1 | -5.05E-01 | 6.37E-02 |  |  |  |
| MYC | -7.17E-01 | 6.38E-02 |  |  |  |
| GEMIN8P4 | -5.56E-01 | 6.50E-02 |  |  |  |
| CENPI | -6.93E-01 | 6.51E-02 |  |  |  |
| PANK3 | -5.46E-01 | 6.57E-02 |  |  |  |
| MKI67 | -7.78E-01 | 6.64E-02 |  |  |  |
| SPAG5 | -6.28E-01 | 6.65E-02 |  |  |  |
| ARMC3 | -8.58E-01 | 6.65E-02 |  |  |  |
| ANLN | -7.67E-01 | 6.67E-02 |  |  |  |
| PLK4 | -6.11E-01 | 6.84E-02 |  |  |  |
| RP11-624L4.1 | -8.93E-01 | 6.85E-02 |  |  |  |
| RP11-739L10.1 | -6.90E-01 | 6.97E-02 |  |  |  |
| ANKEF1 | -5.62E-01 | 7.01E-02 |  |  |  |
| ARTN | -8.40E-01 | 7.08E-02 |  |  |  |
| CYP4F3 | -8.68E-01 | 7.13E-02 |  |  |  |
| HR | -8.23E-01 | 7.14E-02 |  |  |  |
| FEN1 | -5.15E-01 | 7.16E-02 |  |  |  |
| PHLDA2 | -7.25E-01 | 7.17E-02 |  |  |  |
| DTNA | -8.16E-01 | 7.25E-02 |  |  |  |
| H2AFJ | -6.41E-01 | 7.28E-02 |  |  |  |
| GREB1L | -8.58E-01 | 7.29E-02 |  |  |  |
| PPIF | -5.62E-01 | 7.36E-02 |  |  |  |
| NNAT | -6.65E-01 | 7.38E-02 |  |  |  |
| CCND1 | -7.68E-01 | 7.46E-02 |  |  |  |
| KISS1R | -9.17E-01 | 7.51E-02 |  |  |  |
| SLC4A1 | -8.80E-01 | 7.51E-02 |  |  |  |
| KIFC1 | -6.76E-01 | 7.58E-02 |  |  |  |
| RP11-395N3.2 | -8.53E-01 | 7.66E-02 |  |  |  |
| SERPINB5 | -9.16E-01 | 7.66E-02 |  |  |  |
| FAM227A | -5.91E-01 | 7.72E-02 |  |  |  |
| CSTL1 | -9.15E-01 | 7.72E-02 |  |  |  |
| ANKRD13B | -5.88E-01 | 7.75E-02 |  |  |  |
| RAPGEFL1 | -6.85E-01 | 7.76E-02 |  |  |  |
| RAB38 | -7.87E-01 | 7.78E-02 |  |  |  |
| SLC27A2 | -8.68E-01 | 7.83E-02 |  |  |  |
| CDKN3 | -7.47E-01 | 7.90E-02 |  |  |  |
| FJX1 | -7.23E-01 | 7.90E-02 |  |  |  |
| FKBP4 | -5.57E-01 | 8.02E-02 |  |  |  |
| GLRB | -8.10E-01 | 8.41E-02 |  |  |  |
| RP4-816N1.6 | -5.49E-01 | 8.49E-02 |  |  |  |
| REPS2 | -7.86E-01 | 8.52E-02 |  |  |  |
| CCDC78 | -7.88E-01 | 8.61E-02 |  |  |  |
| TTC39A | -6.06E-01 | 8.83E-02 |  |  |  |
| C1orf168 | -8.13E-01 | 8.86E-02 |  |  |  |
| FAM57B | -8.38E-01 | 8.97E-02 |  |  |  |
| SYCP3 | -5.73E-01 | 8.98E-02 |  |  |  |
| GTSE1 | -7.67E-01 | 9.00E-02 |  |  |  |
| HBG1 | -8.53E-01 | 9.02E-02 |  |  |  |
| RP13-608F4.5 | -7.72E-01 | 9.10E-02 |  |  |  |
| AC016700.5 | -7.36E-01 | 9.27E-02 |  |  |  |
| LRTM2 | -8.55E-01 | 9.35E-02 |  |  |  |
| APOBEC3B | -6.21E-01 | 9.43E-02 |  |  |  |
| ACOX2 | -6.93E-01 | 9.43E-02 |  |  |  |
| KIF14 | -7.75E-01 | 9.43E-02 |  |  |  |
| RHOBTB1 | -5.79E-01 | 9.48E-02 |  |  |  |
| ABCA3 | -5.51E-01 | 9.52E-02 |  |  |  |
| NRCAM | -8.49E-01 | 9.52E-02 |  |  |  |
| AURKB | -6.88E-01 | 9.57E-02 |  |  |  |
| FBXO43 | -6.70E-01 | 9.59E-02 |  |  |  |
| ZNF239 | -5.21E-01 | 9.60E-02 |  |  |  |
| MZT2A | -5.58E-01 | 9.65E-02 |  |  |  |
| DSCAML1 | -8.24E-01 | 9.73E-02 |  |  |  |
| SERPINA6 | -8.17E-01 | 9.74E-02 |  |  |  |
| AGPAT6 | -5.32E-01 | 9.77E-02 |  |  |  |
| DEPDC1B | -7.38E-01 | 9.83E-02 |  |  |  |
| SPINK4 | -8.36E-01 | 9.90E-02 |  |  |  |
