# Supplementary material for: Molecular profiling of hormone receptor-positive, HER2-negative breast cancers from patients treated with neoadjuvant endocrine therapy in the CARMINA 02 trial (UCBG-0609)
Source: J Hematol Oncol. 2018 Oct 11;11:124. doi: 10.1186/s13045-018-0670-9 (PMC6180434; doi:10.1186/s13045-018-0670-9)
Supplement: Supplementary file 5 — Table S3. Genes differentially expressed between relapsed tumors and non-relapsed tumors. (DOCX 23 kb) [file 13045_2018_670_MOESM5_ESM.docx]

**Table S3. Genes differentially expressed between relapsed tumors and non-relapsed tumors**

| **Gene ID** | **log2 FC ( relasped/non-relapsed)** | **p-value adjusted** |
| --- | --- | --- |
| **Up-regulated genes** | | |
| SLC26A9 | 3.74E+00 | 2.12E-18 |
| NNAT | 2.11E+00 | 3.68E-08 |
| UGT2B4 | 2.45E+00 | 5.99E-07 |
| SPRR1B | 1.45E+00 | 3.60E-05 |
| ACTL6B | 1.92E+00 | 8.83E-05 |
| MUC19 | 1.97E+00 | 8.83E-05 |
| TNMD | 2.12E+00 | 8.83E-05 |
| AC004947.2 | 1.87E+00 | 1.07E-04 |
| SAA4 | 2.06E+00 | 1.51E-04 |
| HOXA9 | 1.70E+00 | 1.96E-04 |
| RDH5 | 1.72E+00 | 1.96E-04 |
| DES | 2.02E+00 | 1.96E-04 |
| SAA2 | 2.03E+00 | 1.96E-04 |
| SAA2-SAA4 | 2.03E+00 | 1.96E-04 |
| GABRE | 1.69E+00 | 2.91E-04 |
| HCRTR2 | 1.69E+00 | 3.08E-04 |
| LIPE | 1.71E+00 | 3.08E-04 |
| CHGA | 1.80E+00 | 4.63E-04 |
| AC004538.3 | 1.57E+00 | 4.77E-04 |
| THSD7A | 1.58E+00 | 4.95E-04 |
| SELE | 1.76E+00 | 5.27E-04 |
| HOXA10 | 1.50E+00 | 6.76E-04 |
| HOXA10-AS | 1.50E+00 | 7.61E-04 |
| HSPB7 | 1.79E+00 | 7.61E-04 |
| FOXA3 | 1.80E+00 | 7.61E-04 |
| ITGA7 | 1.48E+00 | 7.62E-04 |
| MUC5AC | 1.19E+00 | 8.07E-04 |
| KCNIP2-AS1 | 1.50E+00 | 8.07E-04 |
| KCNIP2 | 1.51E+00 | 8.07E-04 |
| SAA1 | 1.84E+00 | 1.03E-03 |
| HOXA7 | 1.53E+00 | 1.14E-03 |
| FHL1 | 1.64E+00 | 1.14E-03 |
| AQP7P1 | 1.80E+00 | 1.31E-03 |
| SLC25A18 | 1.56E+00 | 1.31E-03 |
| REG4 | 1.72E+00 | 1.49E-03 |
| SLC4A4 | 1.79E+00 | 1.49E-03 |
| PLIN4 | 1.75E+00 | 1.54E-03 |
| MUC5B | 1.74E+00 | 1.72E-03 |
| FAM180B | 1.74E+00 | 1.91E-03 |
| IVL | 1.29E+00 | 2.06E-03 |
| KLF4 | 1.27E+00 | 2.19E-03 |
| C10orf10 | 1.46E+00 | 2.19E-03 |
| AOC3 | 1.51E+00 | 2.59E-03 |
| UGT2B17 | 1.47E+00 | 2.85E-03 |
| ATP1A2 | 1.68E+00 | 2.85E-03 |
| SPNS2 | 9.98E-01 | 4.29E-03 |
| CD34 | 8.57E-01 | 4.58E-03 |
| AC129778.2 | 1.68E+00 | 4.58E-03 |
| PALMD | 1.35E+00 | 5.90E-03 |
| PLIN1 | 1.64E+00 | 6.09E-03 |
| KLB | 1.54E+00 | 6.49E-03 |
| PGA4 | 1.64E+00 | 6.49E-03 |
| ZIC1 | 1.54E+00 | 6.63E-03 |
| CES1 | 1.58E+00 | 6.63E-03 |
| LEP | 1.59E+00 | 6.63E-03 |
| ALDH1L1 | 1.62E+00 | 6.63E-03 |
| GPD1 | 1.64E+00 | 6.63E-03 |
| C7 | 1.64E+00 | 6.63E-03 |
| DGKA | 8.19E-01 | 6.64E-03 |
| FAM129C | 1.56E+00 | 6.64E-03 |
| CIDEC | 1.63E+00 | 6.64E-03 |
| ATP13A5 | 1.54E+00 | 7.19E-03 |
| NEK10 | 1.57E+00 | 7.19E-03 |
| ADRA1A | 1.00E+00 | 7.24E-03 |
| FFAR4 | 1.56E+00 | 7.24E-03 |
| PTGDR | 1.43E+00 | 7.58E-03 |
| CRYAB | 1.38E+00 | 7.61E-03 |
| TRHDE-AS1 | 1.60E+00 | 8.36E-03 |
| GLYAT | 1.55E+00 | 8.90E-03 |
| AQP7 | 1.59E+00 | 9.03E-03 |
| NPR1 | 1.28E+00 | 9.87E-03 |
| SLC19A3 | 1.58E+00 | 1.03E-02 |
| GPIHBP1 | 1.38E+00 | 1.04E-02 |
| GPAM | 1.42E+00 | 1.04E-02 |
| FCER2 | 1.57E+00 | 1.04E-02 |
| ATOH8 | 1.46E+00 | 1.08E-02 |
| TRHDE | 1.56E+00 | 1.12E-02 |
| PEAR1 | 1.03E+00 | 1.25E-02 |
| CIDEA | 1.54E+00 | 1.29E-02 |
| CD300LG | 1.52E+00 | 1.37E-02 |
| PGA5 | 1.52E+00 | 1.37E-02 |
| BMP6 | 1.13E+00 | 1.39E-02 |
| HOXA-AS3 | 1.26E+00 | 1.45E-02 |
| MCAM | 8.60E-01 | 1.46E-02 |
| SELP | 1.47E+00 | 1.46E-02 |
| SCN3A | 1.51E+00 | 1.46E-02 |
| FCRL1 | 1.53E+00 | 1.46E-02 |
| PDE2A | 1.18E+00 | 1.52E-02 |
| MRAP | 6.66E-01 | 1.55E-02 |
| PHKG1 | 9.85E-01 | 1.55E-02 |
| FAM107A | 1.37E+00 | 1.55E-02 |
| CES1P2 | 1.51E+00 | 1.58E-02 |
| CCDC3 | 1.21E+00 | 1.63E-02 |
| SLC14A2 | 1.24E+00 | 1.63E-02 |
| INMT | 1.31E+00 | 1.63E-02 |
| FAM110D | 1.20E+00 | 1.65E-02 |
| RP11-510J16.5 | 1.51E+00 | 1.65E-02 |
| ANGPT1 | 1.26E+00 | 1.67E-02 |
| HSD17B2 | 1.50E+00 | 1.67E-02 |
| RBP4 | 1.50E+00 | 1.67E-02 |
| PCK1 | 1.49E+00 | 1.68E-02 |
| GYS2 | 1.49E+00 | 1.78E-02 |
| BANK1 | 1.42E+00 | 1.81E-02 |
| AQP1 | 1.02E+00 | 1.81E-02 |
| OXER1 | 1.18E+00 | 1.81E-02 |
| ITIH5 | 1.37E+00 | 1.82E-02 |
| NPAS2 | 1.28E+00 | 1.83E-02 |
| HEPN1 | 1.47E+00 | 1.83E-02 |
| KIAA0087 | 1.48E+00 | 1.92E-02 |
| DARC | 1.47E+00 | 1.95E-02 |
| KLHL31 | 1.05E+00 | 1.98E-02 |
| CR2 | 1.39E+00 | 1.98E-02 |
| S100B | 1.46E+00 | 1.98E-02 |
| LINC00341 | 1.04E+00 | 1.99E-02 |
| SEMA3G | 1.03E+00 | 2.24E-02 |
| DTX1 | 1.24E+00 | 2.24E-02 |
| HCAR2 | 1.28E+00 | 2.24E-02 |
| FAM150B | 1.46E+00 | 2.24E-02 |
| ARHGEF15 | 8.48E-01 | 2.26E-02 |
| GPR146 | 7.81E-01 | 2.30E-02 |
| EBF1 | 1.08E+00 | 2.53E-02 |
| VPREB3 | 1.44E+00 | 2.56E-02 |
| ACHE | 1.36E+00 | 2.57E-02 |
| CTA-134P22.2 | 1.42E+00 | 2.59E-02 |
| TACR1 | 1.41E+00 | 2.60E-02 |
| VWF | 8.65E-01 | 2.62E-02 |
| HEPACAM | 1.43E+00 | 2.63E-02 |
| EBF3 | 1.04E+00 | 2.70E-02 |
| CD79B | 1.32E+00 | 2.70E-02 |
| TUSC5 | 1.41E+00 | 2.70E-02 |
| SGCG | 1.43E+00 | 2.70E-02 |
| LHCGR | 1.36E+00 | 2.73E-02 |
| MEOX1 | 1.37E+00 | 2.88E-02 |
| RASGRP2 | 1.14E+00 | 2.88E-02 |
| MUSTN1 | 9.10E-01 | 2.90E-02 |
| NOTCH2 | 6.89E-01 | 2.97E-02 |
| AC009469.1 | 9.14E-01 | 2.97E-02 |
| SLC7A10 | 1.29E+00 | 2.97E-02 |
| AQPEP | 1.40E+00 | 2.97E-02 |
| G0S2 | 1.41E+00 | 2.97E-02 |
| SCARA5 | 1.41E+00 | 2.97E-02 |
| PLCXD3 | 1.41E+00 | 2.97E-02 |
| MS4A1 | 1.41E+00 | 3.14E-02 |
| TMPRSS11E | 1.40E+00 | 3.23E-02 |
| PLXNA4 | 1.26E+00 | 3.27E-02 |
| LINC01140 | 1.31E+00 | 3.40E-02 |
| AKAP12 | 1.19E+00 | 3.41E-02 |
| KLK14 | 1.38E+00 | 3.41E-02 |
| AKR1C1 | 1.38E+00 | 3.41E-02 |
| TRGC1 | 1.35E+00 | 3.42E-02 |
| RP1-28O10.1 | 1.39E+00 | 3.42E-02 |
| AIFM2 | 6.59E-01 | 3.49E-02 |
| PEMT | 8.45E-01 | 3.49E-02 |
| RP11-38L15.3 | 8.64E-01 | 3.49E-02 |
| CLEC1A | 9.24E-01 | 3.49E-02 |
| RP11-439L8.4 | 1.30E+00 | 3.49E-02 |
| PPP1R1A | 1.31E+00 | 3.49E-02 |
| DGAT2 | 1.36E+00 | 3.49E-02 |
| SH2D3C | 7.15E-01 | 3.52E-02 |
| CDH20 | 1.34E+00 | 3.57E-02 |
| CES1P1 | 1.38E+00 | 3.58E-02 |
| TINAGL1 | 1.10E+00 | 3.59E-02 |
| SDPR | 1.30E+00 | 3.59E-02 |
| CXorf36 | 8.03E-01 | 3.60E-02 |
| MLXIPL | 1.32E+00 | 3.60E-02 |
| RP11-439L8.3 | 1.35E+00 | 3.60E-02 |
| CALB2 | 1.32E+00 | 3.75E-02 |
| NOV | 1.27E+00 | 3.86E-02 |
| ACADL | 1.37E+00 | 3.92E-02 |
| RP11-169D4.2 | 1.17E+00 | 4.05E-02 |
| SYT15 | 8.62E-01 | 4.11E-02 |
| PRG4 | 6.10E-01 | 4.25E-02 |
| MALL | 7.97E-01 | 4.30E-02 |
| TNS1 | 8.66E-01 | 4.30E-02 |
| RCAN1 | 1.11E+00 | 4.30E-02 |
| ELMOD3 | 5.08E-01 | 4.36E-02 |
| CAV1 | 1.08E+00 | 4.36E-02 |
| CCDC69 | 1.12E+00 | 4.36E-02 |
| P2RY4 | 1.35E+00 | 4.36E-02 |
| KIF19 | 1.27E+00 | 4.45E-02 |
| C6 | 1.35E+00 | 4.45E-02 |
| CPA4 | 1.28E+00 | 4.46E-02 |
| PGA3 | 1.30E+00 | 4.79E-02 |
| NPAS3 | 1.24E+00 | 4.83E-02 |
| ARR3 | 1.18E+00 | 5.18E-02 |
| PHYHIP | 1.21E+00 | 5.23E-02 |
| RRAD | 1.15E+00 | 5.35E-02 |
| ABCD2 | 1.29E+00 | 5.42E-02 |
| CHRDL1 | 1.32E+00 | 5.61E-02 |
| SPIB | 1.25E+00 | 5.64E-02 |
| HES5 | 1.19E+00 | 5.73E-02 |
| DEFB1 | 1.29E+00 | 5.81E-02 |
| GSN | 7.76E-01 | 5.99E-02 |
| DMRT3 | 9.76E-01 | 5.99E-02 |
| CLIC5 | 1.18E+00 | 5.99E-02 |
| SPRR3 | 8.94E-01 | 6.06E-02 |
| CX3CL1 | 9.12E-01 | 6.06E-02 |
| RASSF4 | 9.62E-01 | 6.06E-02 |
| TNFRSF13B | 1.30E+00 | 6.07E-02 |
| HOXA3 | 1.03E+00 | 6.25E-02 |
| PRODH | 1.28E+00 | 6.27E-02 |
| NGF | 9.54E-01 | 6.39E-02 |
| PKP1 | 1.28E+00 | 6.39E-02 |
| PENK | 1.29E+00 | 6.39E-02 |
| GPX3 | 1.24E+00 | 6.45E-02 |
| CDH5 | 7.64E-01 | 6.46E-02 |
| CCL14 | 1.24E+00 | 6.57E-02 |
| CCL15-CCL14 | 1.24E+00 | 6.57E-02 |
| SFTPA1 | 1.11E+00 | 6.62E-02 |
| SOD3 | 1.15E+00 | 6.70E-02 |
| PYGM | 9.65E-01 | 6.81E-02 |
| MYO7B | 9.75E-01 | 6.81E-02 |
| DGCR6 | 1.16E+00 | 6.81E-02 |
| C8orf34 | 1.21E+00 | 6.81E-02 |
| ROBO4 | 7.32E-01 | 7.19E-02 |
| SOX17 | 7.98E-01 | 7.20E-02 |
| CCL23 | 1.27E+00 | 7.28E-02 |
| POM121L9P | 1.05E+00 | 7.35E-02 |
| S1PR1 | 8.66E-01 | 7.74E-02 |
| HOXA5 | 1.04E+00 | 7.74E-02 |
| ADIPOQ | 1.25E+00 | 7.74E-02 |
| HPD | 1.12E+00 | 7.97E-02 |
| ETS2 | 6.99E-01 | 7.99E-02 |
| HAAO | 9.94E-01 | 8.05E-02 |
| SORBS1 | 1.05E+00 | 8.12E-02 |
| ACVR1C | 1.24E+00 | 8.12E-02 |
| DNASE1L3 | 1.25E+00 | 8.14E-02 |
| SLC29A4 | 9.25E-01 | 8.35E-02 |
| STAT6 | 4.64E-01 | 8.39E-02 |
| S100A7 | 9.68E-01 | 8.61E-02 |
| OLFM4 | 1.21E+00 | 8.72E-02 |
| LPL | 1.23E+00 | 8.87E-02 |
| AADAC | 1.20E+00 | 9.04E-02 |
| RP11-172E9.2 | 1.23E+00 | 9.08E-02 |
| SYN2 | 1.24E+00 | 9.08E-02 |
| C14orf180 | 1.21E+00 | 9.20E-02 |
| AC002480.3 | 1.08E+00 | 9.23E-02 |
| ACACB | 1.04E+00 | 9.27E-02 |
| RP11-415F23.4 | 7.99E-01 | 9.49E-02 |
| NMUR1 | 1.06E+00 | 9.50E-02 |
| PLAC9 | 1.13E+00 | 9.70E-02 |
| CAPN3 | 5.32E-01 | 9.81E-02 |
| RP11-164J13.1 | 5.32E-01 | 9.81E-02 |
| CAV2 | 9.61E-01 | 9.81E-02 |
| HOXB8 | 1.22E+00 | 9.81E-02 |
| TMEM179 | 1.21E+00 | 9.89E-02 |
| **Down-regulated genes** | | |
| HSPA4L | -1.57E+00 | 1.03E-03 |
| MND1 | -1.25E+00 | 1.67E-02 |
| SPATA5 | -7.89E-01 | 1.78E-02 |
| TRIM36 | -1.41E+00 | 1.98E-02 |
| NRIP3 | -1.45E+00 | 2.37E-02 |
| FAM183A | -1.31E+00 | 2.84E-02 |
| PBK | -1.14E+00 | 2.88E-02 |
| KIF18A | -1.06E+00 | 2.97E-02 |
| SOWAHB | -1.20E+00 | 3.31E-02 |
| FSD1L | -1.05E+00 | 3.54E-02 |
| FAM111B | -9.95E-01 | 4.36E-02 |
| C3orf67 | -1.23E+00 | 4.45E-02 |
| CCDC34 | -7.77E-01 | 4.68E-02 |
| TRIM37 | -7.17E-01 | 5.17E-02 |
| ESCO2 | -9.62E-01 | 5.30E-02 |
| MYBL1 | -1.23E+00 | 6.35E-02 |
| CKS2 | -8.26E-01 | 6.39E-02 |
| SLC6A12 | -1.12E+00 | 6.54E-02 |
| ATP6V1H | -6.66E-01 | 6.91E-02 |
| HTR1D | -1.23E+00 | 7.35E-02 |
| CDCA2 | -8.85E-01 | 7.74E-02 |
| WDR63 | -1.19E+00 | 8.12E-02 |
| NRTN | -1.22E+00 | 8.36E-02 |
| SLC12A5 | -1.24E+00 | 8.61E-02 |
| AC012313.1 | -1.24E+00 | 9.23E-02 |
| PLK4 | -7.38E-01 | 9.23E-02 |
| OIP5 | -8.11E-01 | 9.27E-02 |
